# Scalp Topography of Lower Urinary Tract Sensory Evoked Potentials

**DOI:** 10.1007/s10548-020-00796-z

**Published:** 2020-10-16

**Authors:** Stéphanie van der Lely, Thomas M. Kessler, Ulrich Mehnert, Martina D. Liechti

**Affiliations:** Department of Neuro-Urology, Balgrist University Hospital, University of Zürich, Forchstrasse 340, 8008 Zürich, Switzerland

**Keywords:** Brain mapping, Electroencephalography, Electrical stimulation, Evoked potentials, Afferent pathways, Lower urinary tract

## Abstract

Impaired lower urinary tract (LUT) afferents often cause LUT symptoms. Assessment of LUT afferent pathways is possible using bipolar cortical sensory evoked potential (SEP) recordings with the active electrode at the vertex during electrical stimulation in the LUT. This study aimed to investigate the topographical distribution and microstates of lower urinary tract sensory evoked potentials (LUTSEPs) using different stimulation frequencies. Ninety healthy subjects (18–36 years old, 40 women) were randomly assigned to one of five stimulation locations [bladder dome; trigone; proximal, membranous (men only) or distal urethra]. Cycles of 0.5 Hz/1.1 Hz/1.6 Hz electrical stimulation were applied using a custom-made catheter. Cortical activity was recorded from 64 surface electrodes. Marker setting was performed manually on an individual subject-level for the P1, N1, and P2 components of vertex recordings. N1 and P2 topographies presented with central negativities and positivities around the vertex. Regarding topographical distribution, Randomization Graphical User interface (RAGU) analyses revealed consistent frequency effects and microstates for N1/P2. Higher stimulation frequencies resulted in decreasing map strength for P1, N1, and P2. LUTSEP topographies suggest central generators in the somatosensory cortex, which are not detectable in a bipolar set-up. The observed frequency effect indicates fiber refractoriness at higher frequencies. The multichannel approach allows more comprehensive assessment of LUTSEPs and might therefore be sensitive to pathological changes. Examinations in patients with LUT symptoms are needed to further investigate this biomarker.

**Electronic supplementary material:**

The online version of this article (10.1007/s10548-020-00796-z) contains supplementary material, which is available to authorized users.

## Introduction

The brain’s electrical response to ascending sensory input from the lower urinary tract (LUT) conveys important knowledge about the processing of afferent information. This is crucial for the control of LUT function considering that impaired afferent nerve signalling to the brain is involved in LUT dysfunction including symptoms such as urinary urgency, urinary frequency, nocturia and urgency incontinence (Abrams et al. 2002; de Groat and Yoshimura 2009; Fowler et al. 2008). Cortical sensory evoked potentials (SEPs) are used to objectively assess the functionality of the afferent nerve fibers from the periphery up to the cortex and represent the electrical activity of the brain in response to electrical stimulation (Cruccu et al. 2008). By assessing brain electrical activity with high temporal resolution, evoked potentials enable a gradual analysis of neural processing over time. While tibial and pudendal nerve stimulation are well established in clinical practice, the measurement of SEPs in response to repetitive electrical stimulation of the LUT has not yet progressed so far. The latter, however, has proven feasible in healthy subjects (Ganzer et al. 1991; Gerstenberg et al. 1991; Gregorini et al. 2015, 2013; Knüpfer et al. 2018) and patients (Hansen et al. 1989; Sarica et al. 1996) with location-specific lower urinary tract sensory evoked potential (LUTSEP) latencies and amplitudes. Previous studies of our group showed that stimulation at a relatively slow frequency of 0.5 Hz led to more reproducible cortical SEPs in contrast to faster frequencies (Gregorini et al. 2013; Knüpfer et al. 2018; van der Lely et al. 2019b). In addition to age, gender, body weight, and body height, also different stimulation parameters and urine production during stimulation should be considered for LUTSEP analysis (Gerstenberg et al. 1991; Knüpfer et al. 2018; van der Lely et al. 2019a).

Earlier, it was shown that the recording of LUTSEPs with a central active electrode (Cz or Cz-2 cm) and a frontal reference (Fz or Fpz) revealed maximal responses (Badr et al. 1982; Gerstenberg et al. 1991; Gregorini et al. 2013), while amplitudes decreased when recording more anteriorly, laterally or posteriorly. In contrast to single channel recordings of voltage differences between two electrodes, a substantial number of simultaneous recordings are necessary for a suitable assessment of electrical field distribution (Lehmann and Skrandies 1980). In contrast to SEPs following median or tibial nerve stimulation (Kany and Treede 1997), topographical distribution of LUT and pudendal SEPs are less well understood. It was the general goal of this study to provide new information about the central processing of LUT afferent input using analysis of map strength and topographical distribution. In addition, microstate analysis was used to describe the temporal dynamics of the cortical LUTSEP processing steps. We therefore aimed to delineate the cortical topographical distribution of bladder and urethral SEPs (1) in the time course and (2) in dependence of different stimulation frequencies (i.e 0.5 Hz, 1.1 Hz, 1.6 Hz, pulse width = 1.0 ms).

We hypothesized that the different stimulation frequencies reveal similar topographical distribution and temporal dynamics of LUTSEPs with increased map strength for lower stimulation frequencies.

## Materials and Methods

This study was registered at clinicaltrials.gov (identifier NCT02272309). All procedures were approved by the local ethics committee (Kantonale Ethikkommission Zürich) and conducted in accordance with the Declaration of Helsinki. Study data were collected and managed using REDCap (Research Electronic Data Capture software) (Harris et al. 2009). Informed consent was obtained from all individual participants included in the study. All subjects were compensated for their time and effort. We here focus on the multichannel analysis. Detailed information about the general procedure and single channel analysis can be found in van der Lely et al. (2019b) and the supplementary Methods.

### Participants

Healthy subjects were recruited through postings and advertisements on internet platforms. For further details see van der Lely et al. (2019b) and van der Lely et al. (2016). All included subjects fulfilled predefined cut-offs (Table 1).

**Table 1:** Baseline characteristics (n = 90, 40 women)

Baseline characteristics	Women (n = 40)	Men (n = 50)	p value‐gender	p value‐locations
Age [years]	23.5 (18.3–35.8)	23.6 (18.3–34.1)	0.581	0.553
BMI [kg/m^2^]	22.3 (18.3–26.7)	22.8 (19.7–37.2)	0.043^a^	0.928
*3-day bladder diary*				
Micturition frequency per 24 hours	6.5 ± 1.7	5.2 ± 1.9	0.001^a^	0.909
Micturition volume per micturition [mL]	293 (162–718)	339 (209–1057)	0.112	0.534
Fluid intake per 24 h [mL]	2140 (1050–5717)	2115 (783–7953)	0.987	0.484
*Questionnaires*				
ICIQ-FLUTS/MLUTS^b^				
Filling symptoms	1 (0–5)			0.867
Voiding symptoms	0 (0–3)	1 (0–6)		0.178/0.825
Incontinence symptoms	0 (0–2)	0.5 (0–4)		0.539/0.694
IPSS		1 (0–6)		0.611
OAB-q SF				
Symptoms	6 (6–11)	6 (6–16)	0.013^a^	0.340
QoL	13 (13–17)	13 (13–18)	0.188	0.570
HADS				
Anxiety	3.5 (0–7)	3 (0–7)	0.086	0.396
Depression	1 (0–6)	1 (0–6)	0.949	0.558
MoCA	28.5 (26–31)	29 (26–30)	0.802	0.655
*Neuro-urological examination*				
Urogenital sensation (n intact/impaired)	40/0	50/0		
Bulbocavernosus reflex (n intact/impaired)	40/0	49/1		
Anal reflex (n intact/impaired)	40/0	50/0		
Anal sphincter tone (n intact/impaired)	40/0	50/0		
Anal squeeze response (n intact/impaired)	40/0	50/0		
*Free uroflowmetry*				
Voided volume [mL]	448 (161–1243)	393 (95–1195)	0.600	0.394
Maximum flow rate [mL/s]	39.4 (12.4–79.4)	30.6 (11.1–77.4)	0.002^a^	0.227
Post void residual [mL]	1.5 (0–64.5)	3.2 (0–117)	0.190	0.821

*HADS* Hospital Anxiety and Depression Scale, *ICIQ-FLUTS* International Consultation on Incontinence Modular Questionnaire Female lower urinary tract symptoms, *ICIQ-MLUTS* International Consultation on Incontinence Modular Questionnaire Male lower urinary tract symptoms, *IPSS* International Prostate Symptom Score, *MoCA* Montreal Cognitive Assessment, *OAB-q SF* The Overactive Bladder Questionnaire short-form

All subjects fulfilled predefined cut-offs for study inclusion: MoCA score ≥ 26, HADS ≤ 7 each, IPSS ≤ 7, Bladder diary: $\frac{24h urinary frequency}{drinking volume [mL] } \le 0.0045$ with a maximum of 1 × nocturia, mean volume per void > 150 mL and absence of urinary incontinence or urgency. Data are represented as mean ± standard deviation, median (range) or number of subjects (n) if appropriate.

^a^indicates statistical significance p < 0.05.

^b^due to different scoring systems, women and men have not been compared. Supplementary analyses revealed comparable significances when excluding the location membranous urethra. Baseline characteristics stratified for location and gender are shown in van der Lely et al. (2019a).

### Study Design

**Fig. 1:**
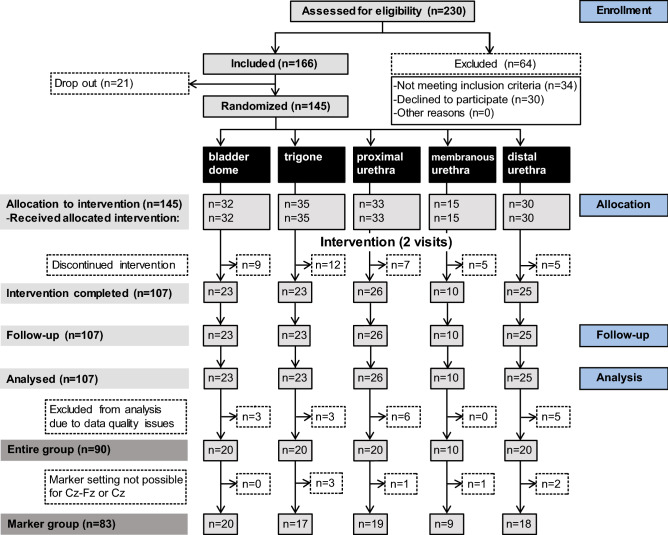
Consort diagram for flow of participants through the study. Reasons for discontinued intervention were: subjects did not complete the assessment (number of subjects: n = 18), catheter could not be placed (n = 14), uncomfortable feeling caused by catheter/stimulation (n = 5), poor health condition (n = 1). Seven subjects were excluded from marker-based analyses since individual marker setting was not possible [TG (n = 3 women), pUR (n = 1 man), mUR (n = 1 man) and dUR (n = 2 women)] on the Cz–Fz, and/or Cz–AvgRef channels. Therefore, only 83 subjects could be included for marker-based analyses. Responder rate was lower for Cz–AvgRef (no markers: LUT n = 5, Pudendus n = 2) compared to Cz–Fz (no markers LUT n = 1, Pudendus n = 1) marker setting (Color figure online)

This was a single centre, randomised, prospective study. All subjects were randomly assigned to one of the following LUT stimulation groups (allocation ratio: 1:1:1:1:1): bladder dome (BD), trigone (TG), proximal urethra (pUR), membranous urethra (mUR, additional location in men), and distal urethra (dUR) (Fig. 1). Here we are investigating the LUTSEP data of a single visit and in comparison to pudendal SEPs using same pulse width (1 ms).

### Recordings

**Fig. 2:**
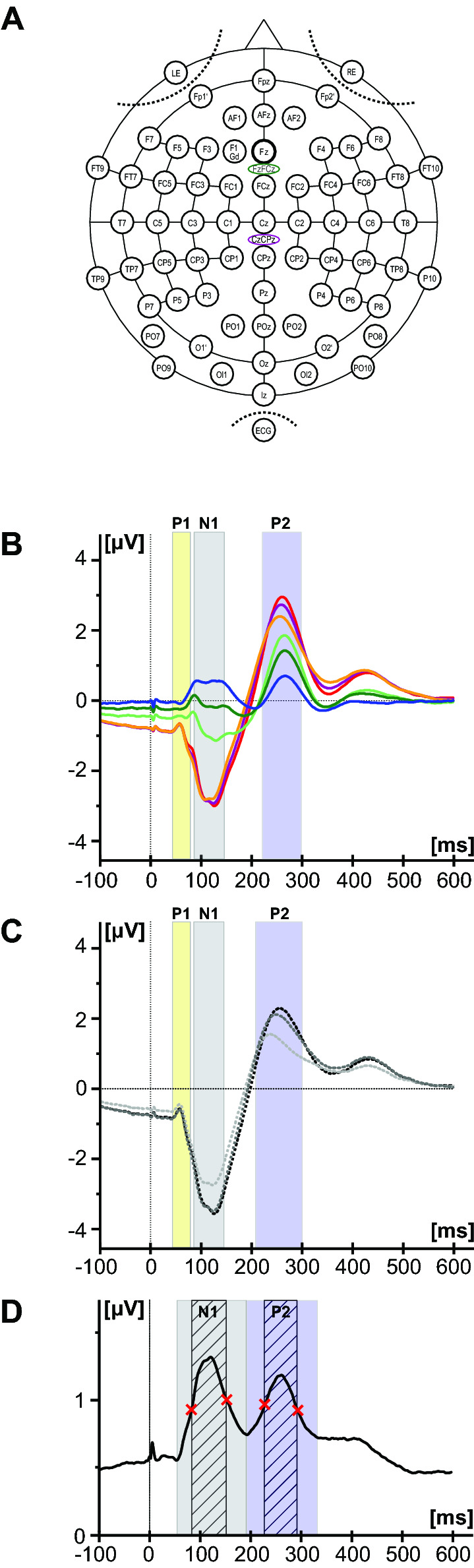
Electrode setup and fixed time windows for topographical analyses of lower urinary tract sensory evoked potentials. **a** Electrodes placed according to an extended international 10–20 system. In addition, two interpolated channels CzCPz and FzFCz are indicated. **b** Sensory evoked potentials (n = 240, all frequencies, all locations without membranous urethra) of the central electrodes Cz, CzCPz and CPz are shown in red, violet and orange against average reference (AvgRef). Potentials for Fz, FzFCz and FCz are shown in blue, dark green and light green. Time windows (mean ± 2 standard deviation) based on the individually set markers of Cz–AvgRef recordings are shown for P1 (yellow), N1 (grey) and P2 (blue). **c** Sensory evoked potentials of the difference channels Cz–Fz, CzCPz–Fz and CzCPz–FzFCz are displayed in black, dark grey and light grey dashed lines. Time windows (mean ± 2standard deviation) based on the individually set markers of Cz–Fz recordings are shown for P1 (yellow), N1 (grey) and P2 (blue). **d** Global field power time windows are indicted based on the start and end points of the N1 (grey) and P2 (blue) component and based on the inflection points (striped). The non-baseline corrected mean global-field power (GFP) curve is shown in black (n = 240, all frequencies, all locations without membranous urethra) with the inflection points indicated by the red crosses (Color figure online)

The electroencephalography (EEG) was continuously recorded from 64 Ag/AgCl surface electrodes with Fz as recording reference and comprised of a cap-based extended international 10–20 montage [Fig. 2a; Easy Cap, Easy Cap GmbH, Herrsching, Germany (Klem et al. 1999)]. The ground electrode was placed at F1 position. For other electrode positions, see supplementary methods. Electrode impedances were kept below 20 kΩ by using abrasive electrolyte gel.

BrainVision Recorder (BrainProducts, Gilching, Germany) and BrainAmp amplifier (Brain Products, Gilching, Germany) were used for a continuous recording and amplification of the signals from the scalp electrodes. The data was digitized at a sampling rate of 5000 Hz and filtered using an analogue filter between 0.016 and 1000 Hz.

### Data Processing and Analyses

Data was pre-processed and analyzed using BrainVision Analyzer2 (Version: 2.0; Brainproducts GmbH, Munich, Germany). The applied filters included a bandpass (0.5–70 Hz; infinite impulse response filters; Butterworth zero-phase shift filter; 24 dB/Oct) and 50 Hz Notch filter. EEG was transformed to average reference (AvgRef, see supplementary methods). Additional channels CzCPz (spherical coordinates: radius = 1, theta = 11,  phi = − 90) and FzFCz (radius = 1, theta = 34, phi = 90) were calculated by spline interpolation (order 4, degree = 10, Fig. 2a,b). Corresponding difference channels were calculated: Cz–Fz, CzCPz–Fz (clinically established recording channel for pudendal SEPs), CzCPz–FzFCz (evoked potentials shown in Fig. 2c).

Subsequently, ocular correction and semiautomatic artefact rejection (± 100 µV) excluding eye blinks, muscle artefacts and technical artefacts was performed and verified by visual inspection.

Continuous EEG was segmented into epochs starting from 100 ms prior to the stimulus to 600 ms post-stimulus (200 ms for pudendal SEP data). For each frequency and participant, artefact free segments were averaged for individual marker setting and group statistics. Group averages across all subjects, and for women and men, separately, were computed for each frequency and stimulation location. In addition, LUTSEPs were also analysed using a baseline correction after segmentation relative to the 53 to 3 ms pre-stimulus time period to account for any pre-stimulus offset.

Individual marker setting was performed manually on recordings of Cz–Fz and Cz–AvgRef. The following criteria had to be fulfilled to set markers: a) overlapping waveform between odd and even SEP averages (= stable SEPs) and b) identifiable P1, N1, P2 LUTSEP respectively P40, N50, P65, N85 pudendal SEP components. Within the same subject, corresponding SEP components were chosen across frequencies. After marker setting, peak latencies, amplitudes and peak-to-peak amplitudes (P1N1 and P2N1 respectively P40N50 and P65N85) were used for analysis. The responder rate was calculated as the percentage of recordings that resulted in a stable SEP with possible marker setting.

#### Time Points and Time Windows for Topographic Analyses

On the one hand, topographic analyses were based on the peak amplitude at the time points of the individually set markers (P1, N1 and P2 for LUT stimulations and P40, N50, P65, N85 for pudendal stimulations, respectively) of the Cz–Fz and Cz–AvgRef channels. On the other hand, mean amplitude was calculated for time windows defined based on the group mean peak latencies (± 2 standard deviations (SD)) of the individually set P1 (Cz–Fz: 43–79 ms; Cz–AvgRef: 43–79 ms), N1 (Cz–Fz: 87–147 ms; Cz–AvgRef: 88–148 ms), and P2 (Cz–Fz: 210-302 ms; Cz–AvgRef: 220–300 ms) markers (n = 222) of the difference channels. In addition, time windows for specific SEP components were chosen in an unbiased way independent from specific channels (Albrecht et al. 2005; Eberhard-Moscicka et al. 2016; Meyer et al. 2007) using the global field power (GFP) as a global measure of the map strength (root mean square of all voltages in a map) representing the SD across all channels (Fig. 2d). The GFP minima reflect the start and end points of the LUTSEP components N1 (54–194 ms) and P2 (194–336 ms), while no GFP peak was visible for component P1. Due to the rather large time windows potentially confounded by adjacent SEP components, we alternatively analysed more narrow time windows between GFP inflection points (N1: 85–154 ms; P2: 228–294 ms).

**Fig. 3:**
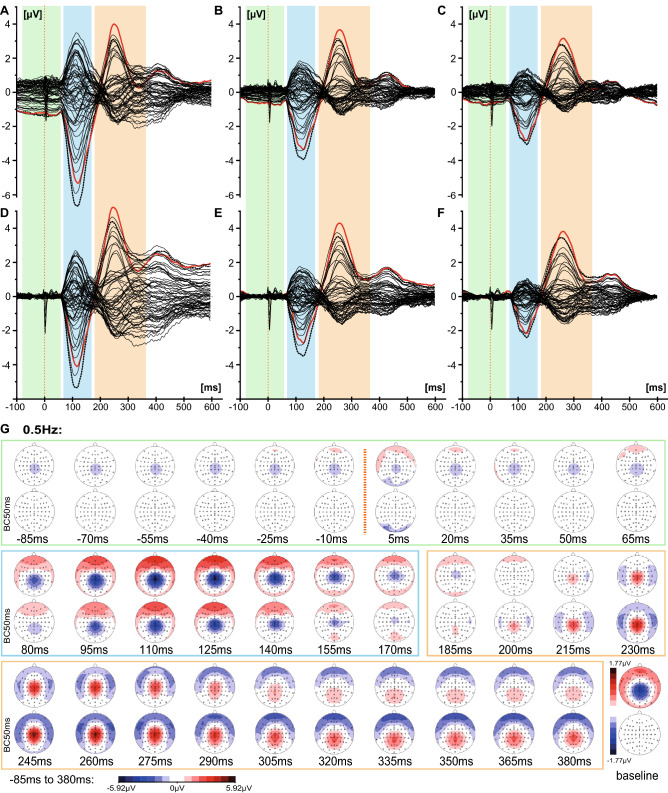

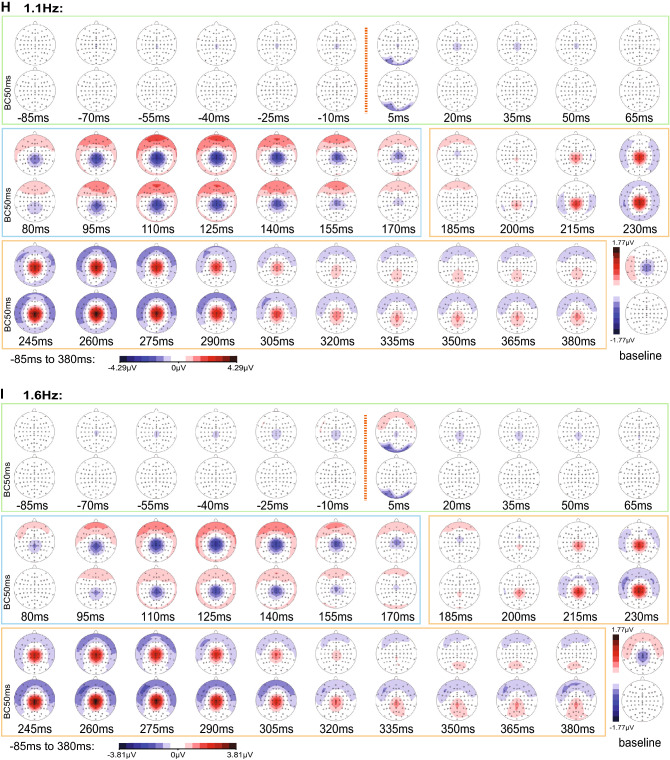
Butterfly plots and time courses of EEG topographies for the location bladder dome. Butterfly plots (−100 to 600 ms) are shown for non-baseline corrected (**a**–**c**) and baseline corrected data (**d**–**f**) of the three stimulation frequencies (0.5 Hz: left, 1.1 Hz middle, 1.6 Hz: right, for all: average of 20 subjects). The recordings from Cz–Fz are indicated by the black dashed line and from Cz–AvgRef by the red line. Time courses of topographical maps are shown at 15 ms time intervals from −85 to 380 ms (average of 20 subjects) for non-baseline corrected data and baseline corrected data of the three stimulation frequencies (**g**–**i**). The maps are subdivided into three sections: −85 to 65 ms (includes pre-stimulus, P1) framed in light green; 80 to 170 ms (N1) framed in light blue; 185 to 380 ms (P2) framed in orange. The time point of the stimulus is indicated by an orange dotted line. The last map represents the mean topography of the baseline time window (−53 to −3 ms) for non-baseline and baseline corrected data (Color figure online)

For a visualisation of the scalp distribution over time, topographic voltage maps of the group averaged LUTSEPs were derived from −85 ms pre-stimulus to 380 ms post-stimulus at an interval of 15 ms (Fig. 3 & supplementary Figs. S1–S4). Since pudendal SEP components are shorter oscillating at higher frequencies, topographic voltage maps were visualized every 7.5 ms from 5.0 ms to 192.5 ms.

### Statistical Analysis

Statistical analyses were performed using RStudio (Version 1.1.453, Boston, MA, U.S.A.) and Randomization Graphical User interface (RAGU) for statistical analyses of multichannel event-related EEG data (Koenig et al. 2011). Alpha level was set at 0.05 for all statistical analyses.

For demographics, means and SDs or median and range (minimum–maximum), where appropriate, were calculated for continuous variables. For dichotomous variables, percentages were reported. Normal distribution of the data was tested using Shapiro–Wilk test and by visual inspection of histogram and qq-plots. To check for gender and location differences of descriptive variables, unpaired Welch’s t-test (normally distributed data) or Mann–Whitney U tests (non-normally distributed data) and one-way ANOVAs (normally distributed data) or Kruskal–Wallis tests (non-normally distributed data) were conducted.

#### Statistics Using Single Channel Marker Information

Cz-Fz marker information was analysed using linear mixed models (LMMs) and default settings of 0.5 Hz stimulation as comparators to the other stimulation frequencies. The following subjects’ characteristics were entered as additional independent variables: age, female gender, location (BD, as comparator to other locations), body weight, body height (or BMI instead of body weight and body height), produced urine volume during the corresponding stimulation cycle, absolute stimulation intensity.

Using this modelling set-up, we examined the effect of various measurement settings and subjects’ characteristics on LUTSEP amplitudes and latencies. To account for repeated measurements between subjects, we introduced an indicator variate for study subject as a random factor to the model. Similarly, pudendal SEP data were analysed, except for the independent variable “produced urine volume”.

Latencies and amplitudes from Cz–Fz were compared to Cz–AvgRef using Wilcoxon-signed rank test.

#### Analysis of Scalp Field Data

For topographic comparisons of stimulation frequencies, paired and for locations/gender, unpaired student’s t-tests (t-maps) were calculated. 62 channels were exported for statistical analyses using RAGU (see supplementary methods).

GFP, the parametric assessment of reference-independent map strength, was calculated as a function of time (Koenig and Melie-Garcia 2010; Lehmann and Skrandies 1980). Since GFP is calculated independently of the spatial distribution, it does not provide information about changes in the topographical distribution either as function of time or across conditions. Using randomization statistics (Koenig et al. 2011), GFP was compared between conditions [for LUTSEP analyses: within-subject factor frequency (3) and between-subject factor location (5); for pudendal SEP analyses: between-subject factor: gender (2)].

Additionally, GFP was used to test for consistent neural activation across subjects in a non-randomized manner by running a Topographic Consistency Test (TCT) (Koenig and Melie-Garcia 2010). Time-wise topographic analysis of variance (TANOVA) was performed [for LUT: with frequency (3) and location (5); for pudendal data: with gender (2)] (Koenig et al. 2011; Koenig and Melie-García 2009; Lehmann and Skrandies 1980; Murray et al. 2008) to compare topographical distribution of multichannel SEP data. TANOVAs are independent of map strength since the dataset is normalized by GFP. For an accurate estimate of the significance level, the analyses were computed with 5000 randomization runs (Manly 2007).

GFP and TANOVA analyses were computed once along the entire SEP segment length (LUT −100 to 600 ms, pudendus −100 to 200 ms) and once based on the individual marker information for Cz–Fz and Cz–AvgRef. In addition, supplementary GFP and TANOVA analyses compared Cz–Fz-based peak information to Cz–AvgRef peak information.

Finally, dynamic changes in SEP topographies were classified using microstate analysis (Brandeis et al. 1995; Lehmann and Skrandies 1980; Murray et al. 2008; Pascual-Marqui et al. 1995). In contrast to time-locked analyses, microstate analyses presume that timing, duration and activation strength of stable brain functional states may vary between conditions. Microstates analyses were performed for the SEP time interval starting from 20 to 600 ms (pudendal SEP 20 to 200 ms) after stimulus onset with microstate prototype maps identification based on the so-called atomize and agglomerate hierarchical clustering algorithm (Murray et al. 2008). The optimal number of microstate classes was defined by cross-validation (Koenig et al. 2014). The cross validation procedure was applied 50 times to the dataset, each time randomly dividing the 83 subjects into training and learning dataset (each 41) and thereby testing each number of microstates ranging from 3 to 20. The optimal model was equivalent to the one with the highest mean correlation between the two data sets. As a next step, every microstate class was assigned to the data of the different conditions and groups using randomization statistics (Koenig et al. 2011; Koenig and Melie-García 2009).

## Results

Ninety subjects were included in the analysis (Fig. 1). Baseline characteristics of the subjects are reported in Table 1.

Subjects reported mild, temporary, and self-limited (1–5 days) dysuria after 109 out of 180 assessments (62 out of 90 subjects) and mild, temporary, and self-limited (1–3 days) hematuria after 9 out of 180 assessments (9 subjects out of 90). Otherwise, all subjects tolerated the procedures well and no symptomatic urinary tract infection was reported.

All SEP averages consisted of at least 350 artefact-free segments, except for 2 out of 90 subjects (2 out of 270 data sets), where a minimum of 105 LUTSEP segments was included. For pudendal SEPs, 1 out of 90 subjects showed a lower number of segments with a minimum of 333 segments, but still providing stable SEPs.

### Single Channel LUTSEPs

The LUTSEPs of all locations and frequencies as well as pudendal SEPs showed typical, previously described P1, N1, P2 and P40, N50, P65, N85 deflections, respectively (Gregorini et al. 2015, 2013; Knüpfer et al. 2018; Pelliccioni et al. 2014; van der Lely et al. 2016). More detailed information on single channel bladder and pudendal SEP results can be found in our previous publication (van der Lely et al. 2019b).

The median latencies and peak-to-peak amplitudes based on the individually set markers for Cz–Fz and Cz–AvgRef recordings are shown in Table 2 and 3. Wilcoxon-signed rank test revealed significant differences between the individually set Cz–Fz and Cz–AvgRef marker positions with increased amplitudes for Cz–Fz (see Tables 2 and 3).

**Table 2:** Median latencies of the main SEP components based on the individually set markers on Cz–Fz and Cz–AvgRef channels

		P1		N1		P2	
		Cz–Fz	Cz–AvgRef	Cz–Fz	Cz–AvgRef	Cz–Fz	Cz–AvgRef
BD (n = 20)	0.5 Hz	62.9 (47.0–78.4)	63.1 (47.2–84.8)	117.9 (99.2–139.2)	120.5 (104.0–160.0)^a^	249.6 (209.4–293.4)	256.3 (219.0–285.4)^a^
	1.1 Hz	64.4 (50.2–81.8)	65.6 (51.0–77.2)	117.5 (97.6–144.6)	118.3 (104.0–157.6)	258.3 (215.8–298.6)	257.7 (223.2–299.2)^a^
	1.6 Hz	70.2 (54.6–84.6)	69.0 (54.2–79.4)	118.6 (101.4–151.6)	119.7 (104.6–157.4)	258.3 (218.4–298.6)	263.9 (219.4–298.8)
TG (n = 17)	0.5 Hz	56.2 (51.4–84.0)	56.6 (52.0–74.8)	119.8 (102.2–158.4)	121.2 (99.0–160.4)	246.6 (225.4–282.2)	256.8 (237.4–285.0)^a^
	1.1 Hz	57.2 (52.6–73.8)	57.8 (47.8–68.4)	121.2 (104.4–152.6)	121.4 (104.4–149.2)	259.2 (236.8–289.4)	261.0 (241.8–287.6)^a^
	1.6 Hz	57.6 (50.4–97.6)	57.4 (48.6–97.2)	123.0 (96.0–165.6)	122.8 (99.2–166.0)	250.2 (229.6–301.2)	254.6 (232.6–299.2)^a^
pUR (n = 19)	0.5 Hz	56.6 (48.4–84.0)	57.4 (47.2–83.2)	116.8 (97.2–137.2)	116.0 (98.6–135.6)	248.0 (216.6–290.4)	258.2 (226.2–287.4)
	1.1 Hz	58.6 (50.6–88.2)	58.2 (50.0–87.0)	112.8 (100.4–144.4)	113.8 (97.2–142.2)	263.4 (221.4–284.2)	265.0 (223.8–287.0)^a^
	1.6 Hz	59.4 (50.8–86.8)	59.2 (49.8–83.2)	113.8 (95.6–153.4)	113.6 (96.0–153.6)	256.4 (215.2–297.8)	260.2 (216.4–290.8)
mUR (n = 9)	0.5 Hz	64.2 (42.8–83.6)	64.4 (42.8–79.8)	119.4 (106.2–156.2)	121.0 (106.2–153.6)	281.6 (245.6–296.4)	277.2 (243.2–301.0)
	1.1 Hz	63.8 (57.2–85.2)	63.4 (52.4–83.4)	138.8 (114.6–164.8)	138.6 (114.6–165.8)	284.4 (248.6–317.4)	275.2 (248.2–322.8)
	1.6 Hz	69.8 (43.4–89.0)	71.2 (42.8–89.6)	134.2 (115.8–152.8)	130.2 (114.8–153.0)	279.2 (245.2–332.2)	283.2 (236.8–328.6)
dUR (n = 18)	0.5 Hz	56.9 (46.6–71.4)	56.9 (47.2–71.4)	100.2 (90.8–137.8)	106.3 (92.4–131.2)	256.7 (225.2–313.8)	259.5 (224.6–313.8)
	1.1 Hz	59.9 (47.2–78.4)	59.7 (46.0–78.4)	104.6 (90.8–140.0)	108.4 (92.2–140.8)	256.7 (225.6–318.6)	256.7 (220.6–291.4)
	1.6 Hz	59.6 (48.8–81.6)	59.4 (48.2–81.6)	104.7 (89.2–134.8)	108.5 (90.8–142.0)	247.9 (185.8–299.4)	260.7 (184.4–305.4)

Pud	P40		N50		P65		N85	
	Cz–Fz	Cz–AvgRef	Cz–Fz	Cz–AvgRef	Cz–Fz	Cz–AvgRef	Cz–Fz	Cz–AvgRef
Women (n = 35)	41.8 (36.0–48.0)	41.6 (34.0–47.8)	53.6 (48.4–60.6)	53.0 (48.6–59.6)^a^	66.0 (58.2–78.8)	65.8 (58.0–77.6)	81.0 (71.8–97.4)	81.4 (71.6–99.0)
Men (n = 48)	45.1 (38.4–52.6)	45.3 (35.8–53.2)	58.1 (52.8–64.6)	57.6 (52.6–66.0)^a^	69.7 (60.0–79.4)	69.6 (59.8–78.2)	86.4 (77.2–94.6)	87.4 (75.6–100.0)

n = 83

^a^indicate significant differences in latencies between Cz–Fz and Cz–AvgRef marker information

**Table 3:** Median peak-to-peak amplitudes of the main SEP components based on the individually set markers on Cz–Fz and Cz–AvgRef channels

		P1N1		P2N1	
		Cz–Fz	Cz–AvgRef	Cz–Fz	Cz–AvgRef
BD (n = 20)	0.5 Hz	5.6 (1.4–12.7)	4.9 (0.5–11.1)^a^	9.6 (2.7–20.6)	8.5 (2.9–18.4)
	1.1 Hz	4.4 (0.3–8.1)	3.4 (0.3–5.9)^a^	7.5 (1.7–14.6)	7.2 (1.6–14.7)
	1.6 Hz	2.7 (1.0–8.3)	2.3 (0.5–7.9)^a^	6.0 (1.6–15.2)	6.2 (1.3–14.5)
TG (n = 17)	0.5 Hz	3.9 (1.0–13.6)	3.3 (0.2–8.4)^a^	9.8 (2.5–20.5)	10.1 (2.1–20.6)
	1.1 Hz	3.0 (0.9–8.7)	2.3 (0.2–6.8)^a^	5.7 (2.2–14.2)	6.7 (2.2–13.7)
	1.6 Hz	2.7 (0.8–5.4)	2.4 (0.1–4.8)	4.5 (1.2–11.4)	5.0 (1.2–12.2)^a^
pUR (n = 19)	0.5 Hz	4.3 (0.8–11.7)	3.1 (0.4–11.6)^a^	7.4 (1.6–16.4)	8.2 (1.3–17.8)
	1.1 Hz	2.6 (1.2–8.1)	2.5 (0.3–8.7)	5.1 (1.0–12.9)	4.6 (1.6–17.0)
	1.6 Hz	2.7 (0.7–7.9)	2.2 (0.3–7.7)	5.1 (1.3–13.7)	4.7 (1.4–14.9)^a^
mUR (n = 9)	0.5 Hz	2.6 (-0.1–8.0)	1.8 (-1.1–6.6)^a^	5.6 (2.0–22.7)	4.8 (1.7–22.1)
	1.1 Hz	1.0 (0.3–4.3)	1.2 (0.1–3.3)	2.5 (0.7–12.4)	2.4 (0.7–11.6)
	1.6 Hz	1.3 (0.6–6.3)	1.1 (0.0–4.8)	2.6 (1.1–14.5)	2.2 (1.0–14.0)
dUR (n = 18)	0.5 Hz	3.4 (0.4–10.1)	2.2 (-0.4–7.1)^a^	6.6 (1.8–16.7)	6.0 (1.6–14.9)^a^
	1.1 Hz	2.6 (0.9–7.3)	2.3 (0.3–5.1)^a^	5.0 (1.9–11.5)	5.6 (2.0–11.1)
	1.6 Hz	2.3 (0.3–4.6)	2.1 (0.1–4.0)	3.7 (1.1–8.3)	3.8 (1.3–8.1)

Pud	P40N50		P65N85	
	Cz–Fz	Cz–AvgRef	Cz–Fz	Cz–AvgRef
Women (n = 35)	1.6 (0.0–5.5)	1.8 (-0.3–5.4)	2.3 (0.1–6.5)	2.4 (0.1–5.7)
Men (n = 48)	1.9 (0.3–5.3)	1.7 (0.5–5.4)^a^	2.1 (0.3–6.9)	2.3 (0.1–7.5)

n = 83

^a^indicate significant differences in amplitudes between Cz–Fz and Cz–AvgRef marker information

LMMs revealed significantly lower LUTSEP amplitudes for higher stimulation frequencies while no frequency effect was shown for LUT latencies. Gender had no significant effect on LUTSEPs. In addition, body height or BMI had no significant effect on latencies.

### Time Course of Multichannel LUTSEPs

Multichannel analysis identified two noteworthy peaks across frequencies and locations (Fig. 3a–f, supplementary Figs. S1–S4a-f). Topographically these prominent peaks presented as a centro-parietal negativity (N1, time frame: 80 to 170 ms) and a central positivity (P2, time frame: 185 to 380 ms) (Fig. 3g–i, supplementary Fig. S1–S4 g-i). The latter shows a transition from a central to a centro-parietal positivity within the P2 time frame. Instead of the expected central positivity in the time frame of P1 (visible in the bipolar channel in Fig. 2c), we observed a very weak central positivity or even central negative topography, which starts already prior to stimulus onset. This so-called contingent negative variation (CNV) was detected more prominently for lower stimulation frequencies. Consequently, further LUTSEP analyses refer to baseline-corrected data.

**Fig. 4:**
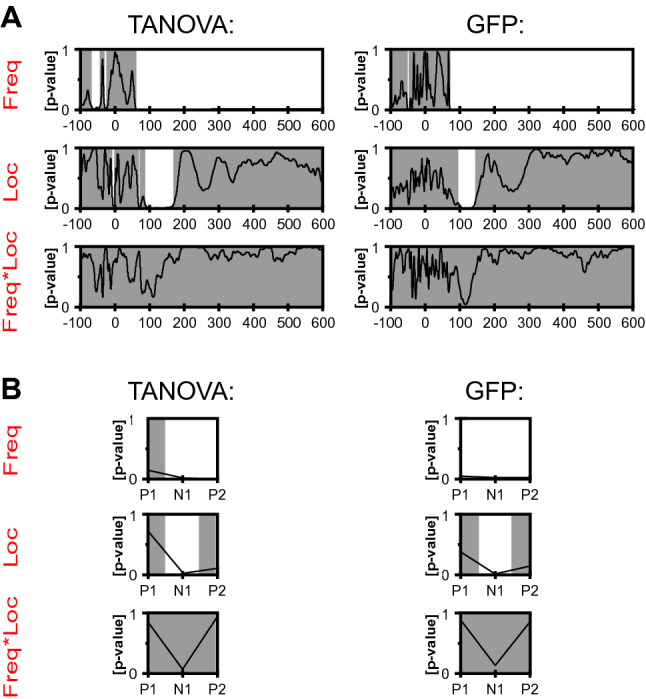
Results of the topographical analysis obtained from baseline-corrected LUTSEP data elicited by using different stimulation frequencies at the five stimulation locations. **a** TANOVA (left) and GFP results (right, n = 83; factors: frequency, stimulation location and frequency*stimulation location) are shown along the entire segment (−100 to 600 ms). The black line marks the p-value as a function of time (x-axis). Grey areas indicate non-significant time intervals while the white areas indicate significant periods between topographic maps of the different factor levels. **b** TANOVA (left) and GFP (right, n = 83; factors: frequency, stimulation location and frequency*stimulation location) are shown for the three Cz–Fz marker positions. The black line marks the p-value for P1, N1, and P2 peak amplitudes (x-axis). Grey areas indicate non-significant while the white areas indicate significant components between topographic maps of the different factor levels. freq frequency, *GFP* global-field power, loc location

Statistical analysis using TANOVA revealed sustained statistically significant topographic differences between frequencies in the time period of about 61 to 600 ms after stimulus (Fig. 4a). For the main effect of location, there was a significant difference in topographies about 89 to 167 ms after stimulus. The millisecond-by-millisecond analysis of the group-averaged GFP waveforms indicating differences in map strength independent of topographic distribution revealed significant differences between frequencies over 70 to 600 ms time periods and significant differences for locations over 101 to 139 ms post-stimulus periods. In addition, the results of the GFP and TANOVA analysis showed no interaction effect between frequency and location (frequency*location).

**Fig. 5:**
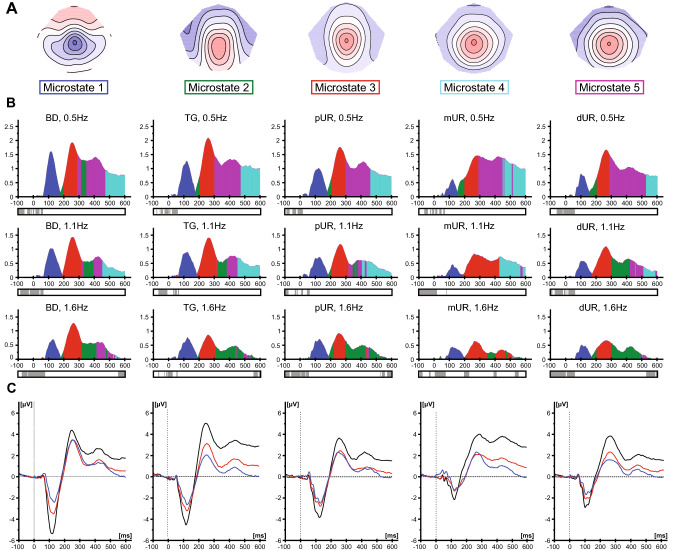
Results of the topographical analysis obtained from baseline-corrected LUTSEP data and wave shapes elicited by using different stimulation frequencies at the five stimulation locations. **a** The five microstate maps obtained from the cross-validation procedure are labelled from 1 to 5 and displayed in sequence of occurrence from left to right (n = 83; orientation: nose up, right is right). Different colors are attributed to different microstate classes. **b** The time course of each microstates map is shown as a function of GFP for the different conditions (frequency, location). Below, results of the topographic consistency test are shown (grey: not significant; white p < 0.05). **c** Grand average cortical SEPs derived from Cz–Fz in response to stimulation with the three stimulation frequencies (baseline-corrected; black 0.5 Hz, red 1.1 Hz, blue 1.6 Hz) at the different locations (total n = 83). *BD* bladder dome, *dUR* distal urethra, *mUR* membranous urethra, *pUR* proximal urethra, *SEP* sensory evoked potential, *TG* trigone (Color figure online)

While statistical analyses showed consistent neural activation after around 70 to 600 ms post-stimulus, cross-validation results identified that the best fitting model for the optimization of the number of microstate classes seemed to be the 5-microstate class solution. The remaining analyses were consequently based on 5(1–5) microstate classes, respectively. Their respective topographies and temporal occurrences are shown in Fig. 5a, b accompanied by the corresponding SEP grand averages (Fig. 5c). Microstate 1, representing a centro-parietal negativity, occurred at the N1 component, while Microstate 3, a central positivity was shown for the P2 peak for all frequencies and locations. While the N1 and P2 components were represented by one microstate map, the microstate of the next positive component varied between conditions.

### Topographic Maps of Single LUTSEP Peaks

**Fig. 6:**
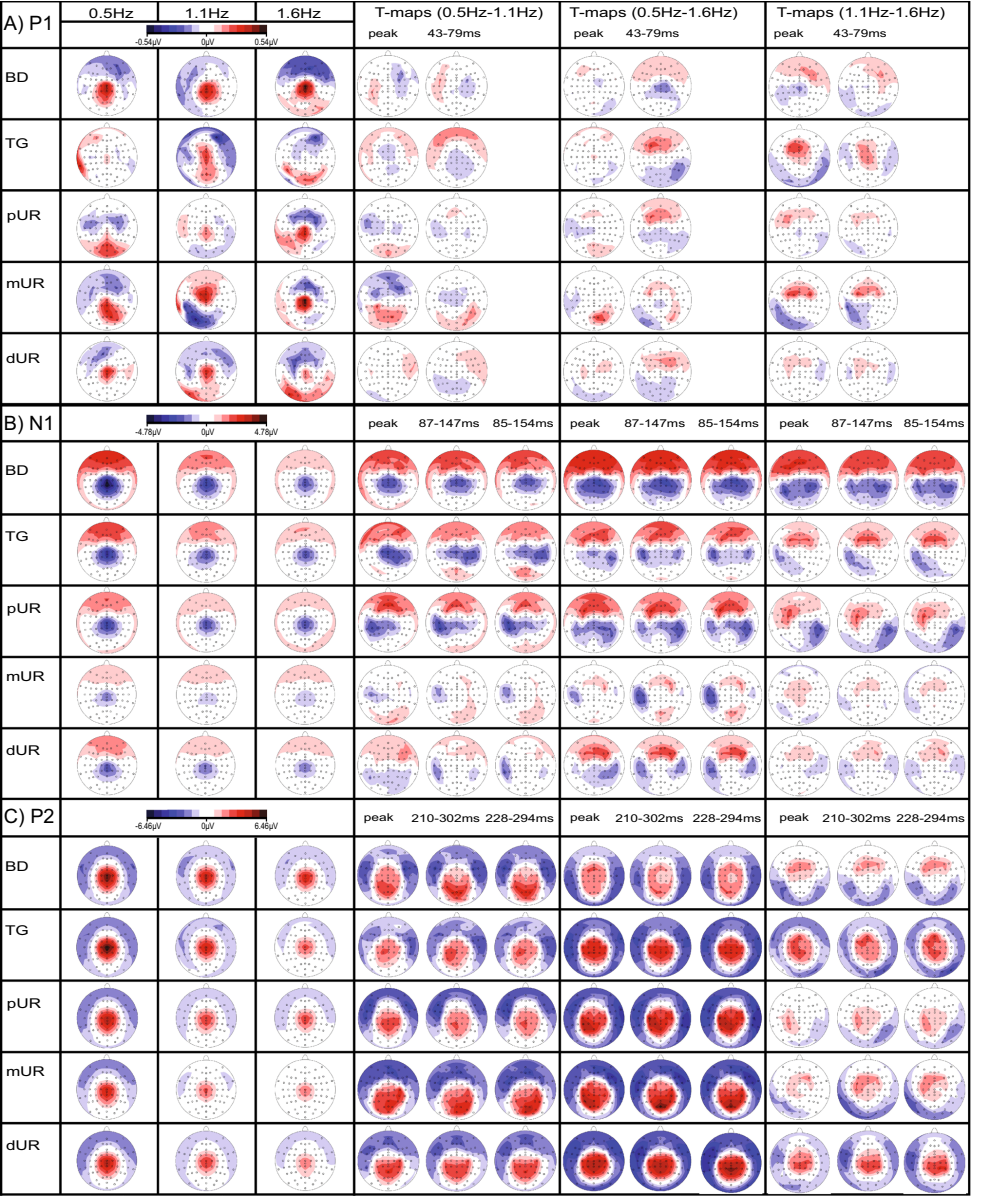
Topographic maps and t-maps of baseline-corrected marker data illustrating frequency effects. Mean topographical P1 (**a**), N1 (**b**), P2 (**c**) maps (based on the individually set Cz–Fz markers) for 0.5 Hz, 1.1 Hz, 1.6 Hz, and per stimulation location: BD (n = 20), TG (n = 17), pUR (n = 19), mUR (n = 9), dUR (n = 18). T-maps are shown for individually set Cz–Fz marker positions (peaks), mean ± 2 SD, and global field power inflection point time windows. Scales in μV/t-values (scale: − 8 to + 8), respectively. T-values of second color grade: statistical trend or significance. *BD* bladder dome, *dUR* distal urethra, *mUR* membranous urethra, *pUR* proximal urethra, *TG* trigone (Color figure online)

Peak analysis based on individually set markers in Cz–Fz and Cz–AvgRef channels revealed no significant differences for topographical distribution and map strength and no interaction for channel with location or frequency. For both approaches, TANOVA showed a significant frequency effect for the topographical distribution of N1 and P2 and a significant location effect for N1 (Fig. 4b). Concerning GFP analyses, again a significant frequency effect for N1 and P2 (exception: P1 reached also significance using Cz–Fz markers) and a location effect for N1 were reported. However, supplementary analyses excluding the location mUR revealed no longer a GFP location effect. The corresponding topographical maps are shown in Fig. 6 in addition to t-maps comparing pairs of different frequencies (Fig. 6) and locations (supplementary Fig. S5). While N1 and P2 showed consistent topographies across conditions, P1 was rather inconsistent with a central positivity only for BD, mUR and dUR. Pair-wise frequency comparisons (t-maps) showed decreasing map strength for higher stimulation frequencies with significantly lower amplitudes over the central areas for N1 and P2. The location mUR showed less differences in map strength between frequencies for N1, while the biggest differences were shown for BD. Similar N1 and P2 frequency comparisons were shown when using fixed time windows (GFP inflection points or mean ± 2SD time) or the individually set Cz–Fz peaks.

### Pudendal SEPs

Pudendal SEP results can be found in the supplementary Results (see also supplementary Fig. S6 & S7).

## Discussion

The main LUTSEP component N1 presented topographically with a centro-parietal negativity followed by a central positivity in the time frame of the P2 peak. P1, however, proved to be the weakest component mainly existing in the bipolar recordings and seemed to be inconsistent when looking at butterfly plots, TCT, GFP and scalp topographies (Figs. 3–6).

We investigated whether frequency and stimulation site-specific LUTSEPs could be differentiated by scalp potential topographies and microstate profiles in healthy subjects. The topographical and microstate results support our hypothesis of increased map strength for lower stimulation frequencies.

Using the standard bipolar Cz–Fz approach, the shape and latencies of LUTSEPs (P1, N1, P2) and pudendal SEPs (P40, N50, P65, N85) correspond well to results of previous studies (Gregorini et al. 2015, 2013; Knüpfer et al. 2018; Pelliccioni et al. 2014).

In detail, N1 component presented topographically as a centro-parietal negativity with a frontal positivity, while the P2 component presented as a central positivity with a circular negative surrounding (Fig. 6). Pudendal SEP topographies averaged across all 90 healthy subjects showed a central negativity for N50 and N85 in women and men while P40 and P65 positivities were less comparable between genders (supplementary Fig. S7). Maxima of the P40 component appeared a bit more posterior compared to the LUTSEP components.

### Frequency Effect

A significant frequency effect was observed for the topographical distribution and map strength of the LUTSEP peaks, both, for continuous data along the entire segment and for individually set marker positions (Cz–Fz and Cz–AvgRef, Fig. 4). In accordance with our hypothesis and the bipolar recording, decreasing map strength was observed for higher stimulation frequencies with significantly lower amplitudes over the central areas for N1 and P2. Following our previous publications on bipolar LUTSEPs, lower stimulation frequencies might be more suitable for stimulation of the LUT slow fibers while higher frequencies might lead to fiber refractoriness (Gregorini et al. 2015, 2013; Knüpfer et al. 2018; van der Lely et al. 2019b). The observed alterations in scalp topographical distribution across conditions could be based on two causes. On the one hand, the conditions that are compared may have the same scalp topographies but differ in the time-distribution of the evoked potential. On the other hand, conditions may differ in the scalp topographies. This is the reason why this analysis was followed by a microstate analysis in order to analyse temporal aspects of electrical LUT stimulation processing. In fact our results show the same microstates across conditions for the N1 and also P2 component, respectively across all conditions, which is indicative that the same underlying neural sources are active (Khanna et al. 2015).

Another, more technical difference between the stimulation frequencies was the observation of a negative shift in the pre-stimulus phase when using slow frequencies (see technical considerations).

### Location Effect

Regarding our exploratory analysis, a significant between-subject location effect in the topographical distribution was observed for the area of N1. Map strength showed as well significant differences but in a more narrow and earlier part of N1. The significant difference in map strength, however, was less stable compared to the changes in topographical distribution since it was no longer significant when excluding the location mUR. Especially BD showed a significantly stronger central negativity (spreading to more posterior electrodes) and anterior positivity for N1 compared to the other locations (see t-maps in supplementary Fig. S5). This is in correspondence with differences in peak-to-peak amplitudes for Cz–Fz with higher amplitudes for BD, TG and pUR (Gregorini et al. 2013; Knüpfer et al. 2018). Significant differences in map strength between locations could be explained by the differential neuronal innervation, which is known to have the highest density at the TG in women and men (Spradling et al. 2015). In accordance, increased map strength was found for TG with decreasing map strength from proximal to distal parts of the urethra. The comparatively large map strength observed for BD needs further investigation. On the other hand, the observed topographical location differences might rather be explained by group differences than anatomical differences.

While LUTSEPs results showed no gender effects, a significant gender effect with shorter peak latencies (P40, N50, P65, N85) was found for pudendal SEPs in women; which is comparable to previous studies (Pelliccioni et al. 2014). This, in addition to the fact that pudendal SEP components are quite narrow and lie close to each other, prevented the application of fixed time windows. Analyses of pudendal SEP topographies showed differential activation of neuronal sources between genders, which may be due to anatomical differences and differential processing known from genital sensation (Cazala et al. 2015).

### Technical Considerations

The channel “Cz” was located in the topographical minima and maxima of N1 and P2 LUTSEP components, which corresponds well with the sensory homunculus location of sensory afferents from midline urogenital structures (Michels et al. 2010). Regarding the positioning of the reference channel, “Fz” proved useful for the N1 component. For P2, at first sight, a more frontally recording position would be ideal, however, practically impossible due to the known eye artefacts that could impact the signal. In contrast to multichannel recordings allowing sophisticated eye-artefact correction, eye artefacts would be a problem when measuring SEPs with only two electrodes.

The observation of the LUTSEP topographies explains why recordings from Cz–Fz showed for example bigger amplitudes compared to CzCPz–Fz and CzCPz–FzFCz. When comparing the mean amplitudes at the individually set marker positions of Cz–Fz to Cz–AvgRef, no significant differences were shown for the topographical distribution and map strength. As expected, the use of an average reference in contrast to Fz reference, leads to reduced amplitudes. This may be explained by the fact that the average reference comes the closest to a neutral site, because, theoretically, activity in opposing dipoles may cancel each other out. If limited to bipolar recording, we conclude that recordings from Cz–Fz are a good solution for LUTSEPs also regarding responder rate, which even proved higher for Cz–Fz compared to Cz–AvgRef.

Using fixed GFP time windows would lead to a big saving of time since one is not dependent on any manual marker setting, however, this was only possible for N1 and P2 and is questionable for patient data with the expected elongated latencies.

The CNV offset was most prominently seen when stimulating with slower frequencies and was not present when stimulating with 3.1 Hz at the pudendal nerve. This CNV is an indicator of arousal processes, expectation, and attention to the experimental task. We assume that the subjects develop a stronger CNV with slower frequencies due to the bigger interstimulus interval. As known from the literature, CNV appears most prominently at the vertex  as a  bilaterally symmetrical topography (Tecce 1972). This central CNV slowly merged with the negativity of the N1 peak. Thereby it suppressed the development of the P1 positivity. Baseline correction of the data permitted a better comparison of the three stimulation frequencies. In future studies the application of a varying interstimulus interval instead of a fixed stimulation frequency could reduce the amplitude of the CNV.

### LUTSEPs in Research and Clinics

While the methods that are currently available for the investigation of LUT afferent nerve function (i.e urodynamic investigation) cannot detect and explain all pathological mechanisms of LUT dysfunction, new diagnostic options are needed. LUTSEP assessment is such an option that has the potential to become an objective marker of afferent nerve function in research and potentially also in clinical practice. However, previous studies complicated the drawing of conclusions due to heterogeneous study populations and measurement settings. This study provides for the first time a LUTSEP evaluation in both gender groups with a systematic comparison of different stimulation frequencies at different locations in the LUT. Our refined LUTSEP protocol may advance the evaluation of viscerosensory afferent pathways in patients with LUT dysfunction and has the potential to serve as a clinical diagnostic tool complementary to standard urodynamic investigations.

In addition to standard SEP recording, the analysis of brain topographies has neurophysiological utility considering that differences between conditions represent changes in the underlying cortical generators, which are not visible when measuring SEPs in a bipolar set-up. Multichannel EEG recordings may help to better differentiate between healthy subjects and patients due to valuable spatial information. Thereby, microstates may allow elucidating coping strategies of patients, as topographic differences may point to regional loci of altered brain function (Griffiths et al. 2005). Such changes in brain function reflected in topographic differences may result from alterations in subcortical modulating influences or relationships between multiple cortical generators of SEPs. Microstate analysis could represent an ideal method to compare different time points of patient data and healthy subjects without the need of elaborate and time-consuming marker setting. We suppose that topographical analyses may find its application as biomarker for treatment effects as well as regenerative processes. In addition to the current set-up, segmental spinal recordings and complementary imaging modalities (i.e fMRI), as well as source localizations might shed light on the underlying sources and anatomical pathways. An advantage of EEG topography analysis compared to other imaging techniques such as positron emission topography, magnetic resonance imaging and magnetoencephalography is that it is a non-invasive, inexpensive technique that has the highest temporal resolution and does not use ionizing radiation.

### Limitations

Study design with separate groups per location complicates the interpretation of location effects. Tailored towards frequency effects, the investigation of location effects was not the intended goal of the present study. Having several locations in addition to three different frequencies would be too many subsequent stimulations in the same subject which may lead to irritations of the LUT and reductions in the subjects’ adherence. Once the LUTSEP method has been optimized, we plan to further investigate different LUT locations within-subjects.

The current results are derived from healthy young subjects only. Considering that aging increases the risk of bladder problems, for patient studies, age should be included as covariate and the age range of the controls should be extended according to the age of the patient group.

## Conclusions

Repetitive electrical stimulation in the healthy LUT leads to consistent LUTSEP microstates and cortical topographies with a centro-parietal negativity for N1 and a central positivity for P2 components across locations. These results would be compatible with a main central generator in the somatosensory cortex. For bipolar recordings, Cz–Fz seems to be a reasonable choice, also considering the high responder rate based on manual peak detection, but it needs to be further investigated in patients. In addition, a clear frequency effect was shown with reduced map strength for higher stimulation frequencies indicating refractoriness of the fibers in response to faster frequencies. Compared to conventional bipolar recordings, the multichannel approach allows a more comprehensive investigation comprising pathological findings in areas outside the maximum peak activity. Moreover, microstate analysis is independent from semi-subjective marker setting and can deal with variations in timing. This is a huge advantage when applied in patients, who are expected to have elongated latencies. Microstates may also allow elucidating coping strategies of patients, as topographic differences may point to regional loci of altered brain function together with changes in underlying cortical generators of SEPs. Further studies in patients with LUT symptoms are required to define the application field of this biomarker.

## Electronic supplementary material

Below is the link to the electronic supplementary material.

Supplementary file1 (pdf 41180 kb)

## Abbreviations

BD: Bladder dome
CNV: Contingent negative variation
dUR: Distal urethra
EEG: Electroencephalography
GFP: Global field power
HADS: Hospital Anxiety and Depression Scale
ICIQ-FLUTS: International Consultation on Incontinence Modular Questionnaire Female lower urinary tract symptoms
ICIQ-MLUTS: International consultation on Incontinence Modular Questionnaire Male lower urinary tract symptoms
IPSS: International Prostate Symptom Score
LMM: Linear mixed models
LUT: Lower urinary tract
LUTSEP: Lower urinary tract sensory evoked potential
M: Men
MoCA: Montreal Cognitive Assessment
mUR: Membranous urethra
OAB-q SF: The Overactive Bladder Questionnaire short-form
pUR: Proximal urethra
RAGU: Randomization Graphical User interface
REDCap: Research Electronic Data Capture
SD: Standard deviation
SEP: Sensory evoked potential
TANOVA: Topographic analysis of variance
TCT: Topographic Consistency Test
TG: Trigone
W: Women
