# Supplementary material for: Scalp Topography of Lower Urinary Tract Sensory Evoked Potentials
Source: Brain Topogr. 2020 Oct 16;33(6):693–709. doi: 10.1007/s10548-020-00796-z (PMC7593393; doi:10.1007/s10548-020-00796-z)
Supplement: Supplementary file 1 — Supplementary file1 (pdf 41180 kb) [file 10548_2020_796_MOESM1_ESM.pdf]

Scalp topography of lower urinary tract sensory evoked potentials

Journal name: Brain Topography

Authors: Stéphanie van der Lely, Thomas M. Kessler, Ulrich Mehnert*, Martina D. Liechti* (*joint senior authors)

Corresponding author: Dr. sc. ETH Martina Liechti
Department of Neuro-Urology
Balgrist University Hospital
University of Zürich
Forchstrasse 340
8008 Zürich, Switzerland
Phone: +41 44 386 37 21
Fax: +41 44 386 39 09
martina.liechti@balgrist.ch

Supplementary Materials and Methods

Electrode Positions according to the International 10-20 System with additional Electrodes:

Electrode positions were chosen according to the international 10-20 system with additional electrodes at AF1/2, AFz, C1/2/5/6, CP1/2/3/4/5/6, CPz, F5/6, FC1/2/3/4/5/6, FCz, FPz, FT7/8/9/10, Iz, Oz, P5/6, PO1/2/9/10, POz, TP7/8/9/10 (Fig. 2a). For more even coverage, the four electrodes O1', O2', Fp1' and Fp2' were placed 15% more laterally to Oz or Fpz, respectively. The electrodes Ol1' and Ol2' were placed to the left and to the right of the midline, halfway between Oz and Iz, to provide better coverage of the occipital scalp distributions.

Electrooculogram was recorded by two electrodes placed 1cm behind and below the outer canthus of each eye in addition to electrocardiogram (Fig. 2a).

Channels included into Average Reference:

In addition to Fz recording reference, average reference (AvgRef) includes: AF1, AF2, AFz, C1, C2, C3, C4, C5, C6, CP1, CP2, CP3, CP4, CP5, CP6, CPz, Cz, F3, F4, F5, F6, F7, F8, FC1, FC2, FC3, FC4, FC5, FC6, FCz, Fp1, Fp2, Fpz, FT10, FT7, FT8, FT9, Iz, O1, O2, OI1, OI2, Oz, P3, P4, P5, P6, P7, P8, PO1, PO10, PO2, PO9, POz, Pz, T7, T8, TP10, TP7, TP8, TP9.

Channels exported for Statistical Analyses performed in RAGU:

AF1, AF2, AFz, C1, C2, C3, C4, C5, C6, CP1, CP2, CP3, CP4, CP5, CP6, CPz, Cz, F3, F4, F5, F6, F7, F8, FC1, FC2, FC3, FC4, FC5, FC6, FCz, Fp1, Fp2, Fpz, FT10,

FT7, FT8, FT9, Fz, Iz, O1, O2, OI1, OI2, Oz, P3, P4, P5, P6, P7, P8, PO1, PO10, PO2, PO9, POz, Pz, T7, T8, TP10, TP7, TP8, TP9.

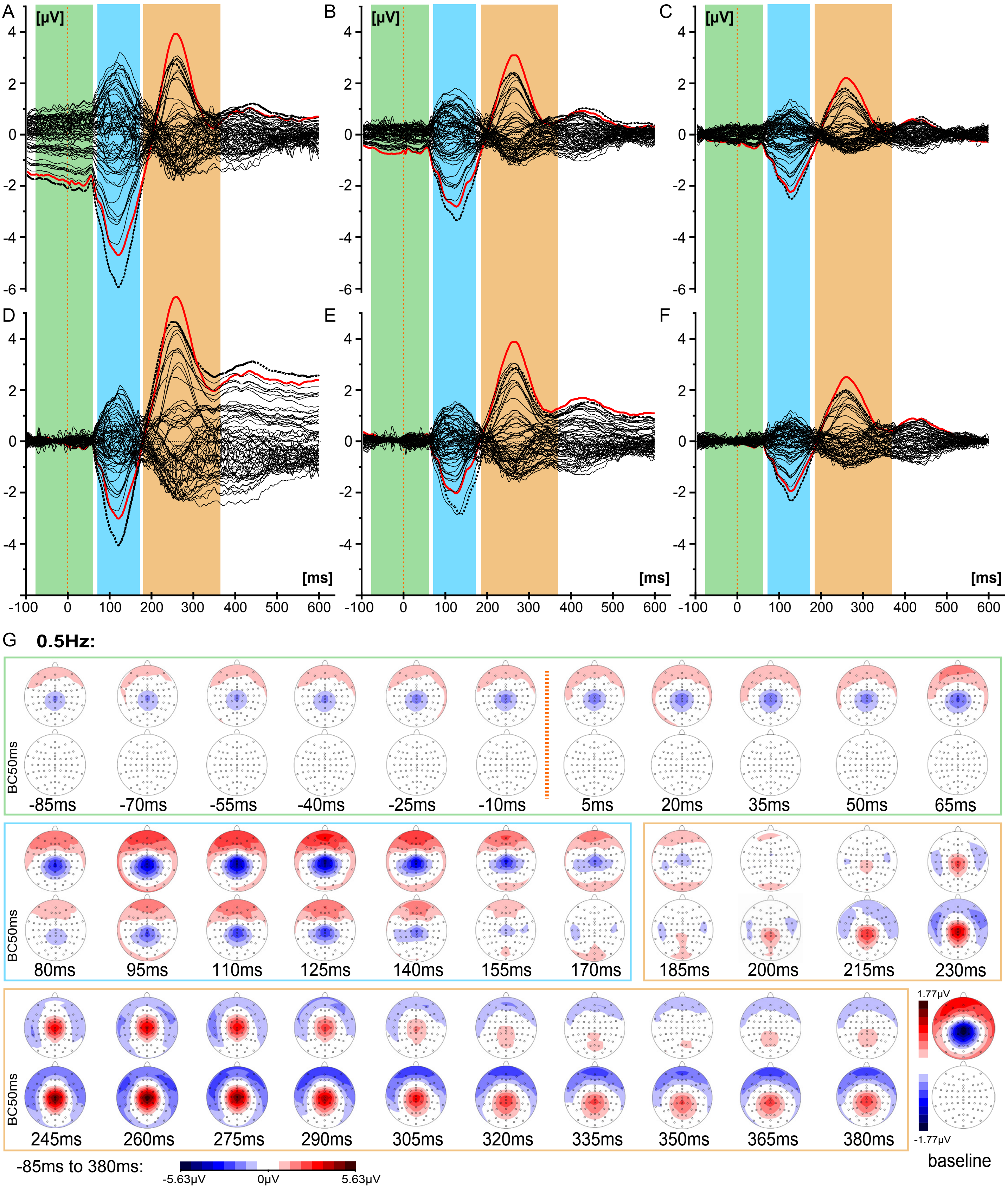
A
B
C
D
E
F
[µV]
[ms]
4
2
0
-2
-4
-6
-100
0
100
200
300
400
500
600
G
0.5Hz:
BC50ms
-85ms
-70ms
-55ms
-40ms
-25ms
-10ms
5ms
20ms
35ms
50ms
65ms
80ms
95ms
110ms
125ms
140ms
155ms
170ms
185ms
200ms
215ms
230ms
245ms
260ms
275ms
290ms
305ms
320ms
335ms
350ms
365ms
380ms
1.77µV
-1.77µV
baseline
-85ms to 380ms:
-5.63µV
0µV
5.63µV

H **1.1Hz:**

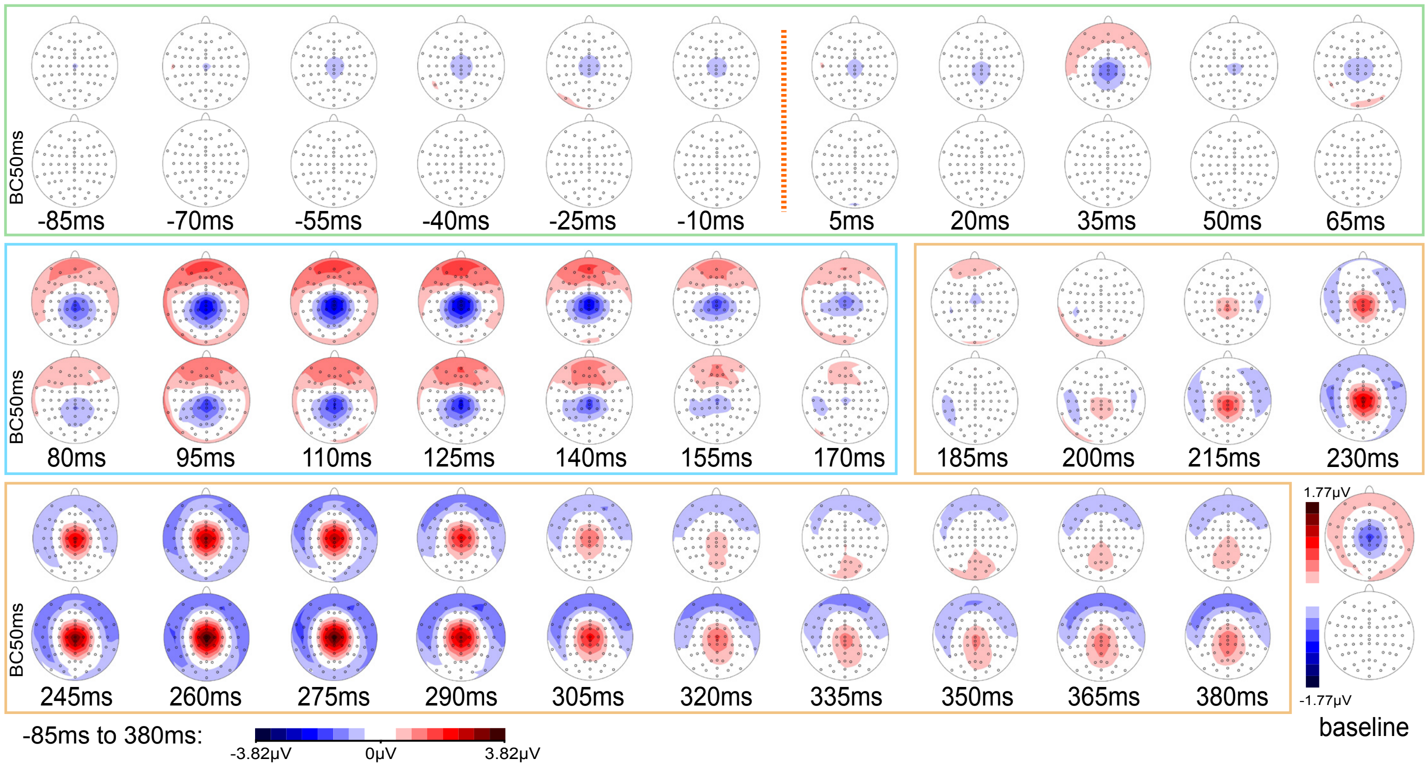

I **1.6Hz:**

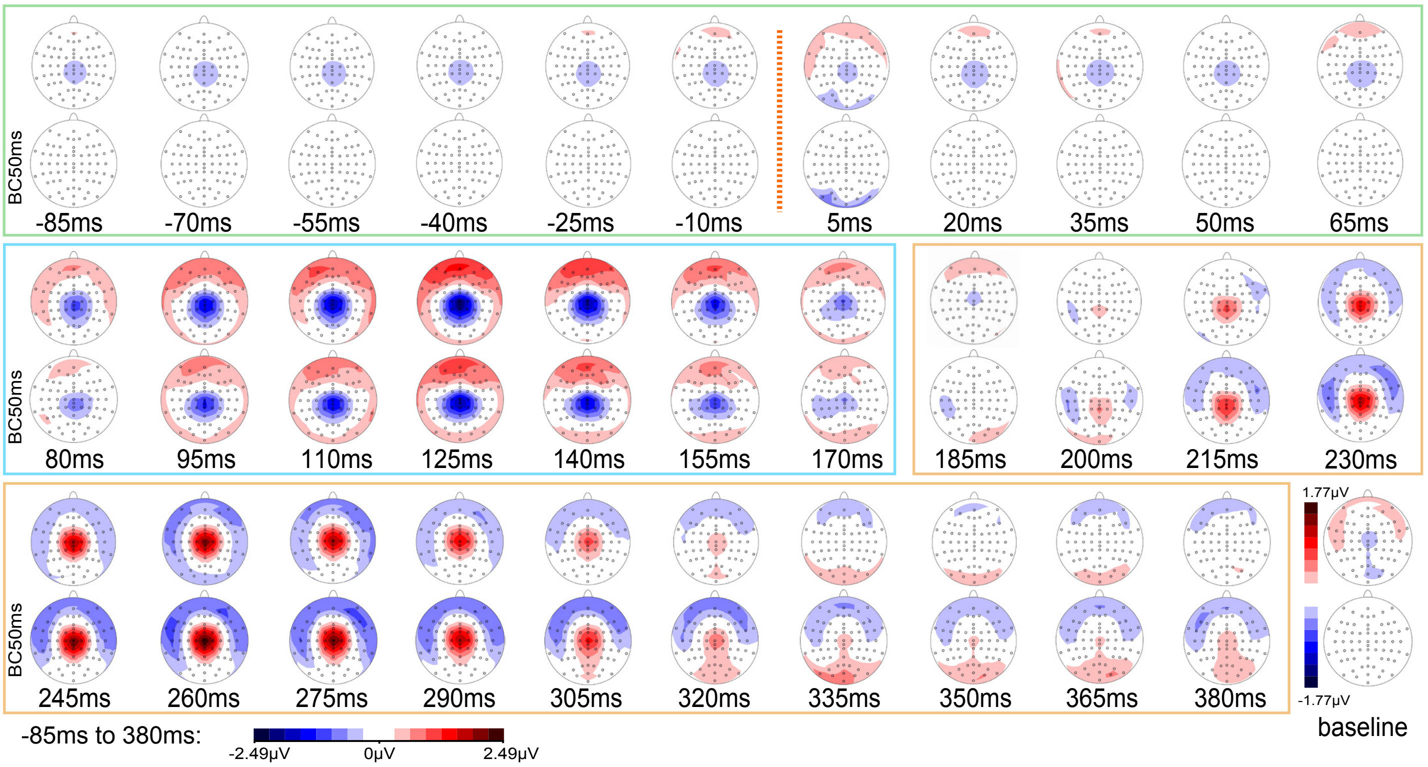

Fig. ESM1 Butterfly plots and time courses of EEG topographies for the location trigone. Butterfly plots (-100 to 600ms) are shown for non-baseline corrected (**a-c**) and baseline corrected data (**d-f**) of the three stimulation frequencies (0.5Hz: left, 1.1Hz: middle, 1.6Hz: right, for all: average of 20 subjects). The recordings from Cz-Fz are indicated by the black dashed line and from Cz-AvgRef by the red line. Time courses of topographical maps are shown at 15ms time intervals from -85 to 380ms (average of 20 subjects) for non-baseline corrected data and baseline corrected data of the three stimulation frequencies (**g-i**). The maps are subdivided into three sections: -85 to 65ms (includes pre-stimulus, P1) framed in light green; 80 to 170ms (N1) framed in light blue; 185 to 380ms (P2) framed in orange. The time point of the stimulus is indicated by an orange dotted line. The last map represents the mean topography of the baseline time window (-53 to -3ms) for non-baseline and baseline corrected data

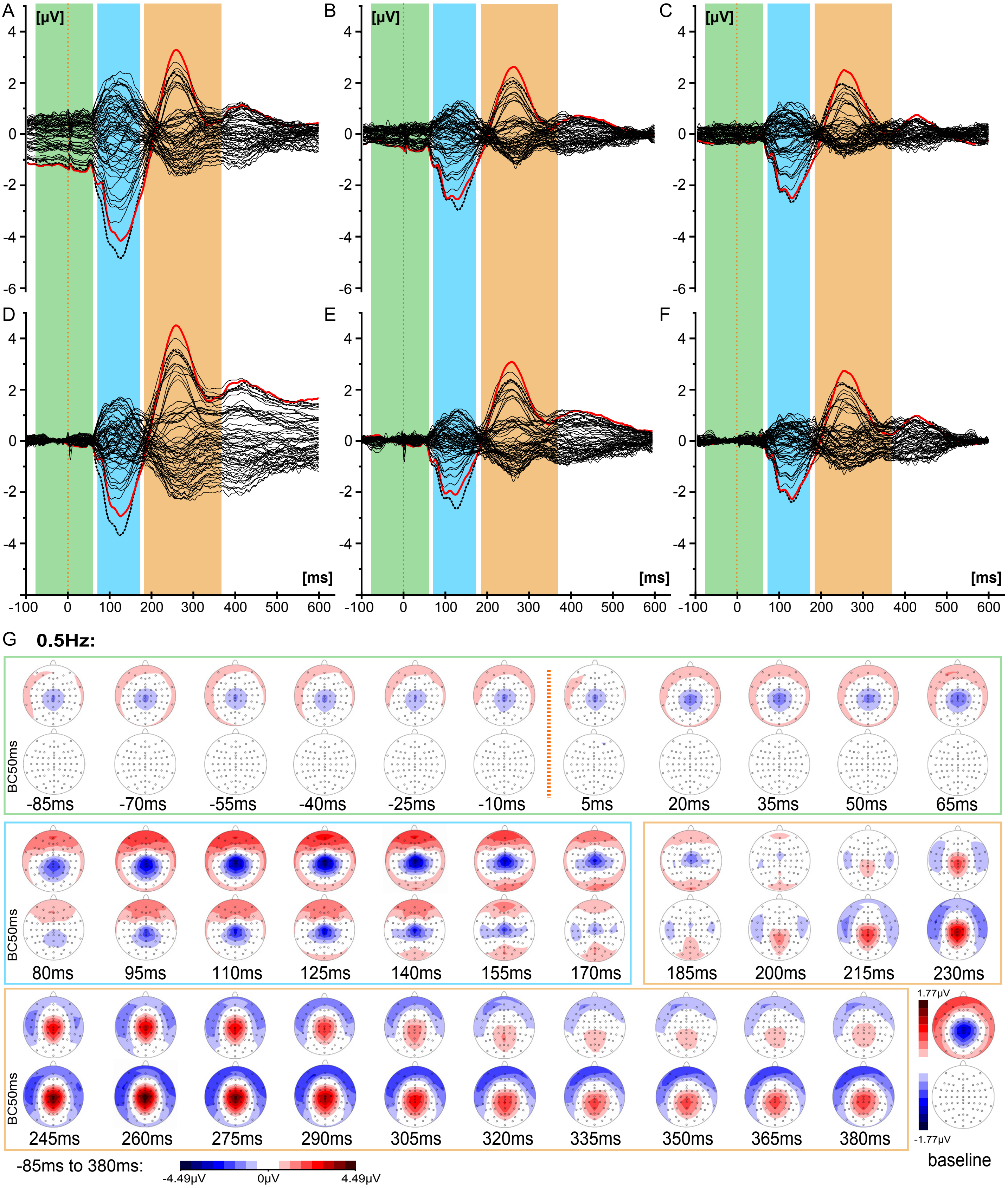

A
B
C
D
E
F
[μV]
[ms]
G
0.5Hz:
BC50ms
-85ms
-70ms
-55ms
-40ms
-25ms
-10ms
5ms
20ms
35ms
50ms
65ms
80ms
95ms
110ms
125ms
140ms
155ms
170ms
185ms
200ms
215ms
230ms
245ms
260ms
275ms
290ms
305ms
320ms
335ms
350ms
365ms
380ms
1.77μV
-1.77μV
baseline
-85ms to 380ms:
-4.49μV
0μV
4.49μV

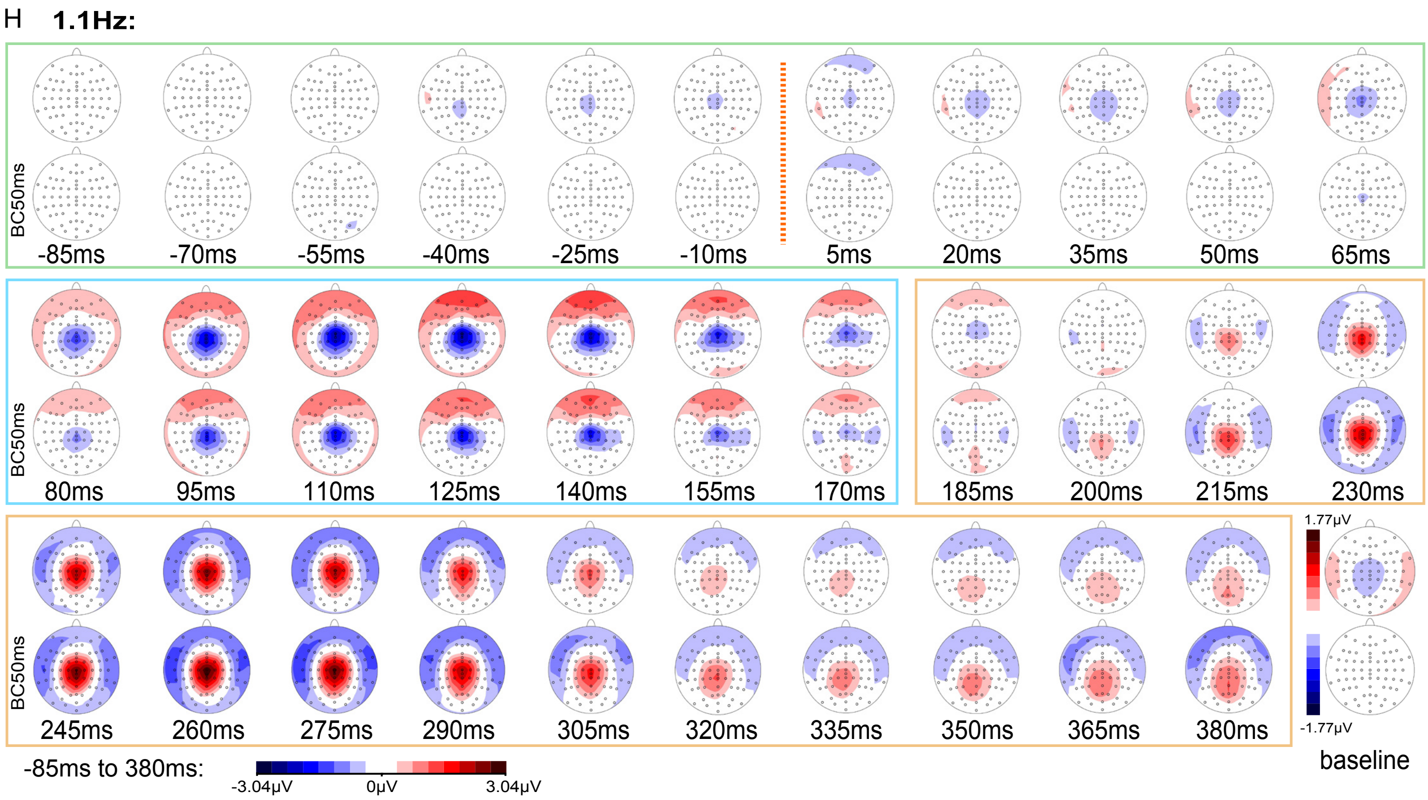

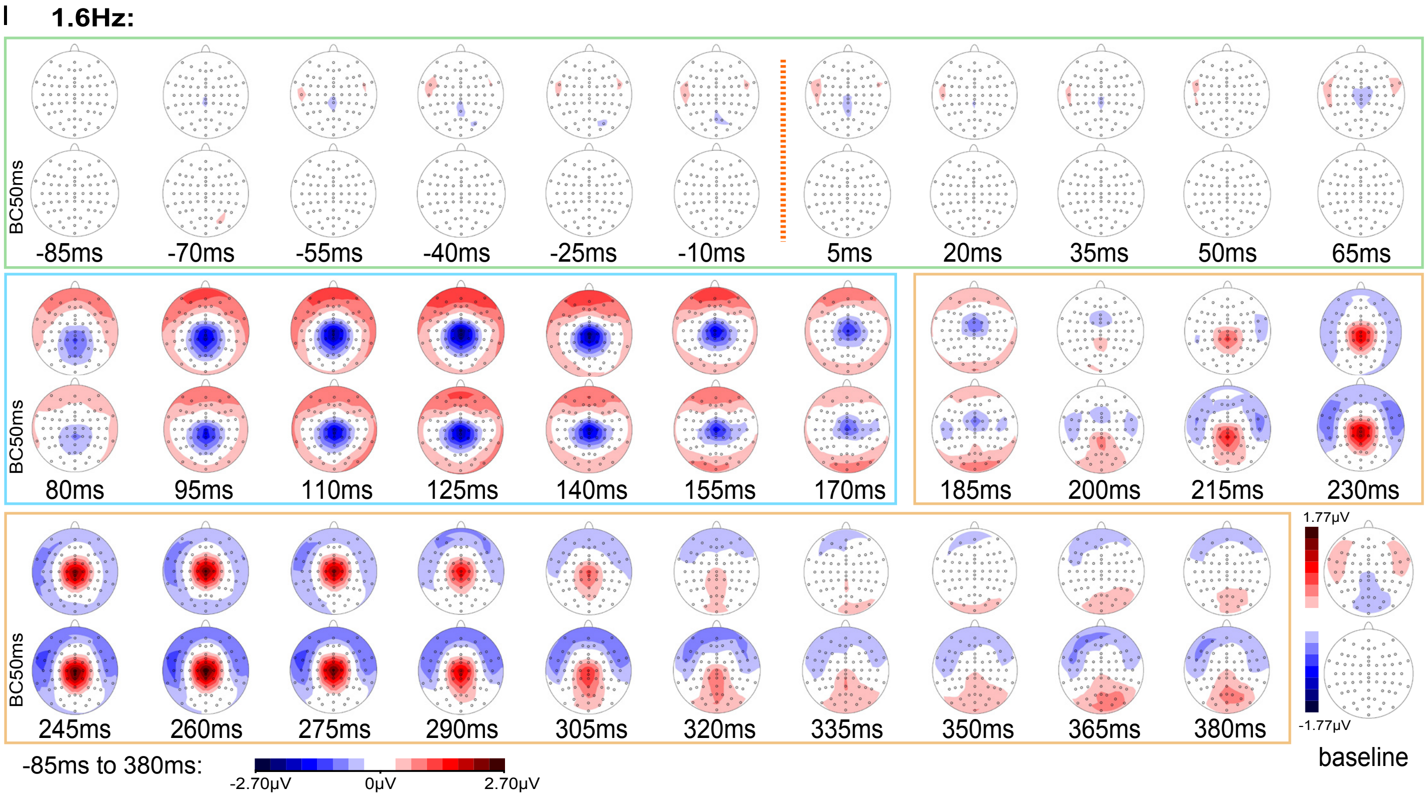

Fig. ESM2 Butterfly plots and time courses of EEG topographies for the location proximal urethra. Butterfly plots (-100 to 600ms) are shown for non-baseline corrected (**a-c**) and baseline corrected data (**d-f**) of the three stimulation frequencies (0.5Hz: left, 1.1Hz: middle, 1.6Hz: right, for all: average of 20 subjects). The recordings from Cz-Fz are indicated by the black dashed line and from Cz-AvgRef by the red line. Time courses of topographical maps are shown at 15ms time intervals from -85 to 380ms (average of 20 subjects) for non-baseline corrected data and baseline corrected data of the three stimulation frequencies (**g-i**). The maps are subdivided into three sections: -85 to 65ms (includes pre-stimulus, P1) framed in light green; 80 to 170ms (N1) framed in light blue; 185 to 380ms (P2) framed in orange. The time point of the stimulus is indicated by an orange dotted line. The last map represents the mean topography of the baseline time window (-53 to -3ms) for non-baseline and baseline corrected data

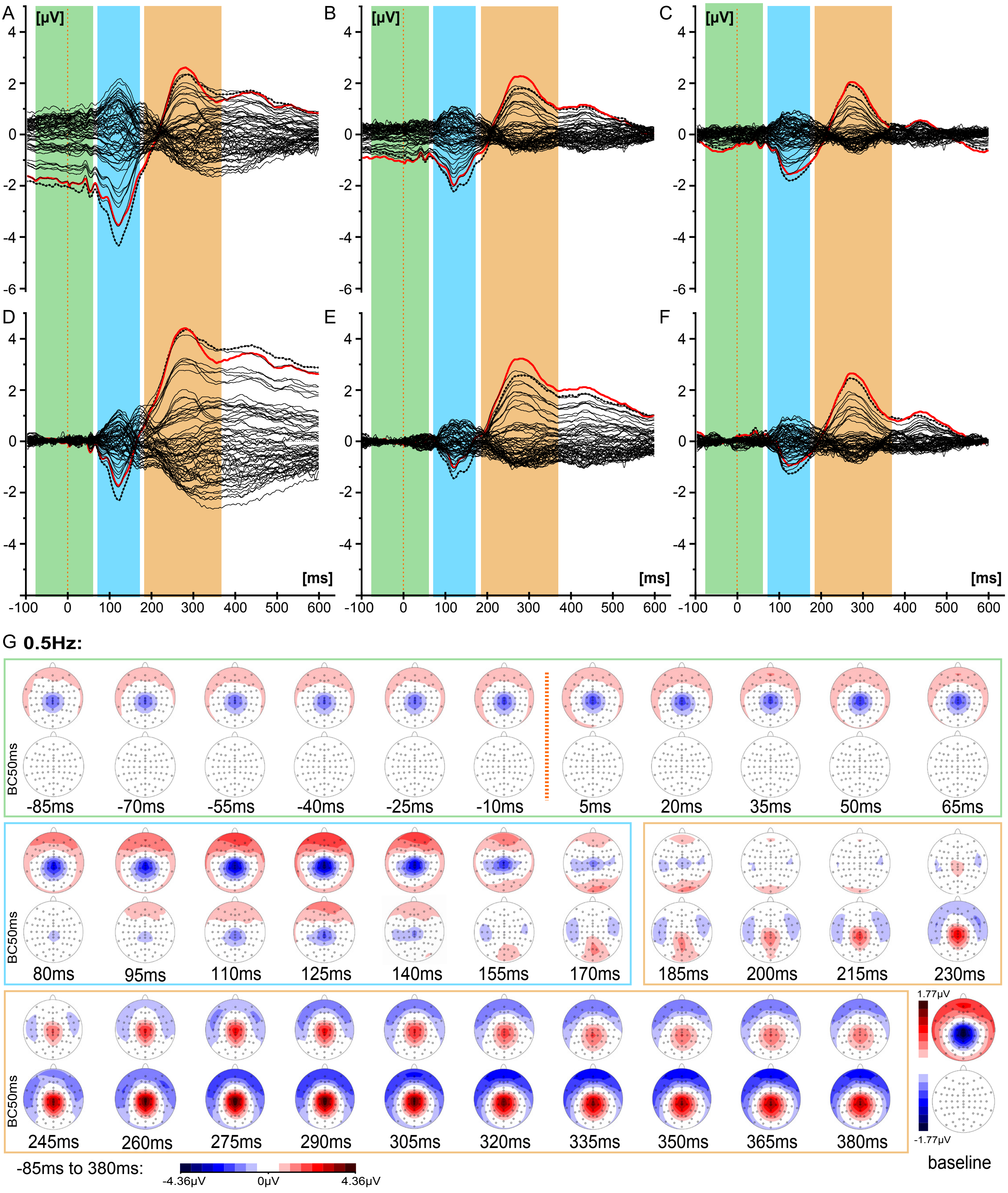

A
B
C
D
E
F
[µV]
[ms]
-100
0
100
200
300
400
500
600
G 0.5Hz:
BC50ms
-85ms
-70ms
-55ms
-40ms
-25ms
-10ms
5ms
20ms
35ms
50ms
65ms
80ms
95ms
110ms
125ms
140ms
155ms
170ms
185ms
200ms
215ms
230ms
245ms
260ms
275ms
290ms
305ms
320ms
335ms
350ms
365ms
380ms
1.77µV
-1.77µV
baseline
-85ms to 380ms:
-4.36µV
0µV
4.36µV

H **1.1Hz:**

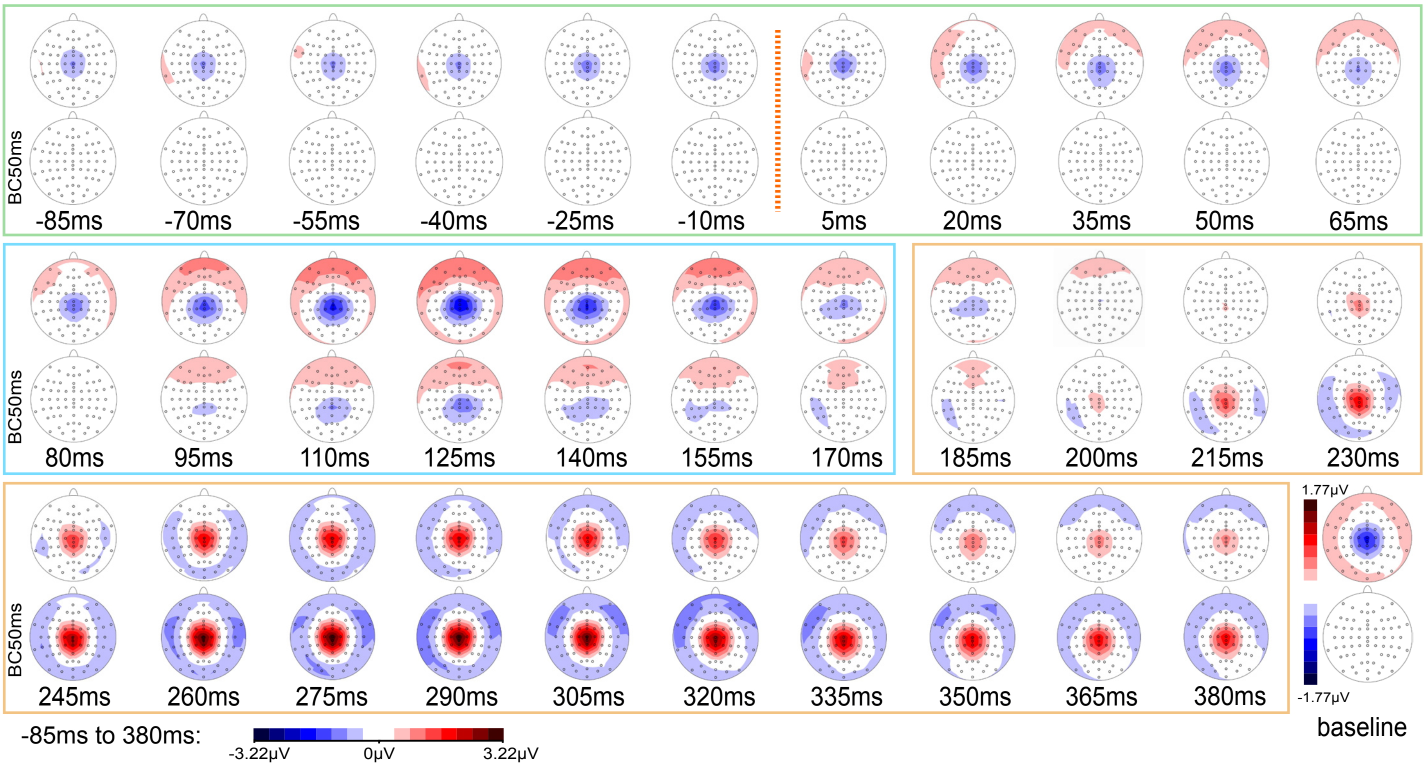

I **1.6Hz:**

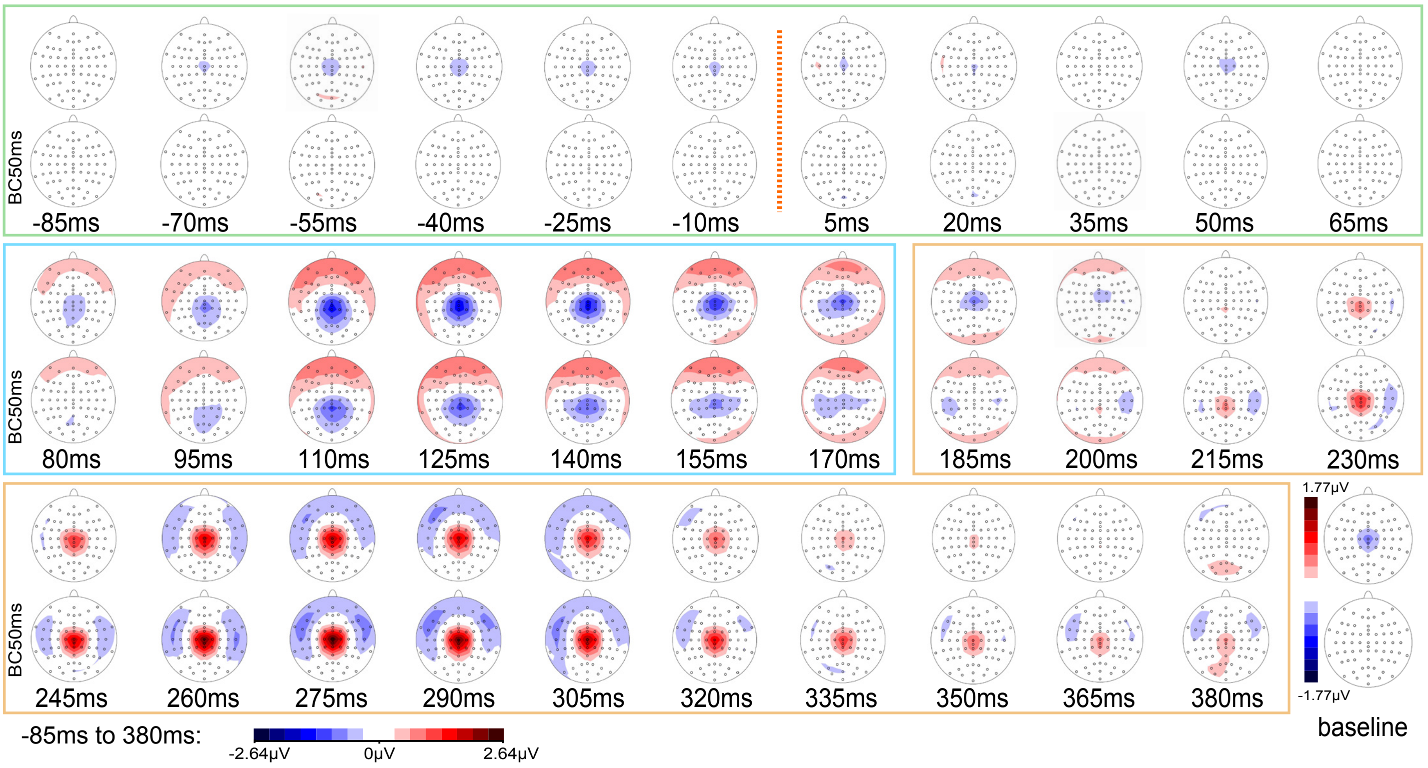

Fig. ESM3 Butterfly plots and time courses of EEG topographies for the location membranous urethra. Butterfly plots (-100 to 600ms) are shown for non-baseline corrected (**a-c**) and baseline corrected data (**d-f**) of the three stimulation frequencies (0.5Hz: left, 1.1Hz: middle, 1.6Hz: right, for all: average of 10 subjects). The recordings from Cz-Fz are indicated by the black dashed line and from Cz-AvgRef by the red line. Time courses of topographical maps are shown at 15ms time intervals from -85 to 380ms (average of 10 subjects) for non-baseline corrected data and baseline corrected data of the three stimulation frequencies (**g-i**). The maps are subdivided into three sections: -85 to 65ms (includes pre-stimulus, P1) framed in light green; 80 to 170ms (N1) framed in light blue; 185 to 380ms (P2) framed in orange. The time point of the stimulus is indicated by an orange dotted line. The last map represents the mean topography of the baseline time window (-53 to -3ms) for non-baseline and baseline corrected data

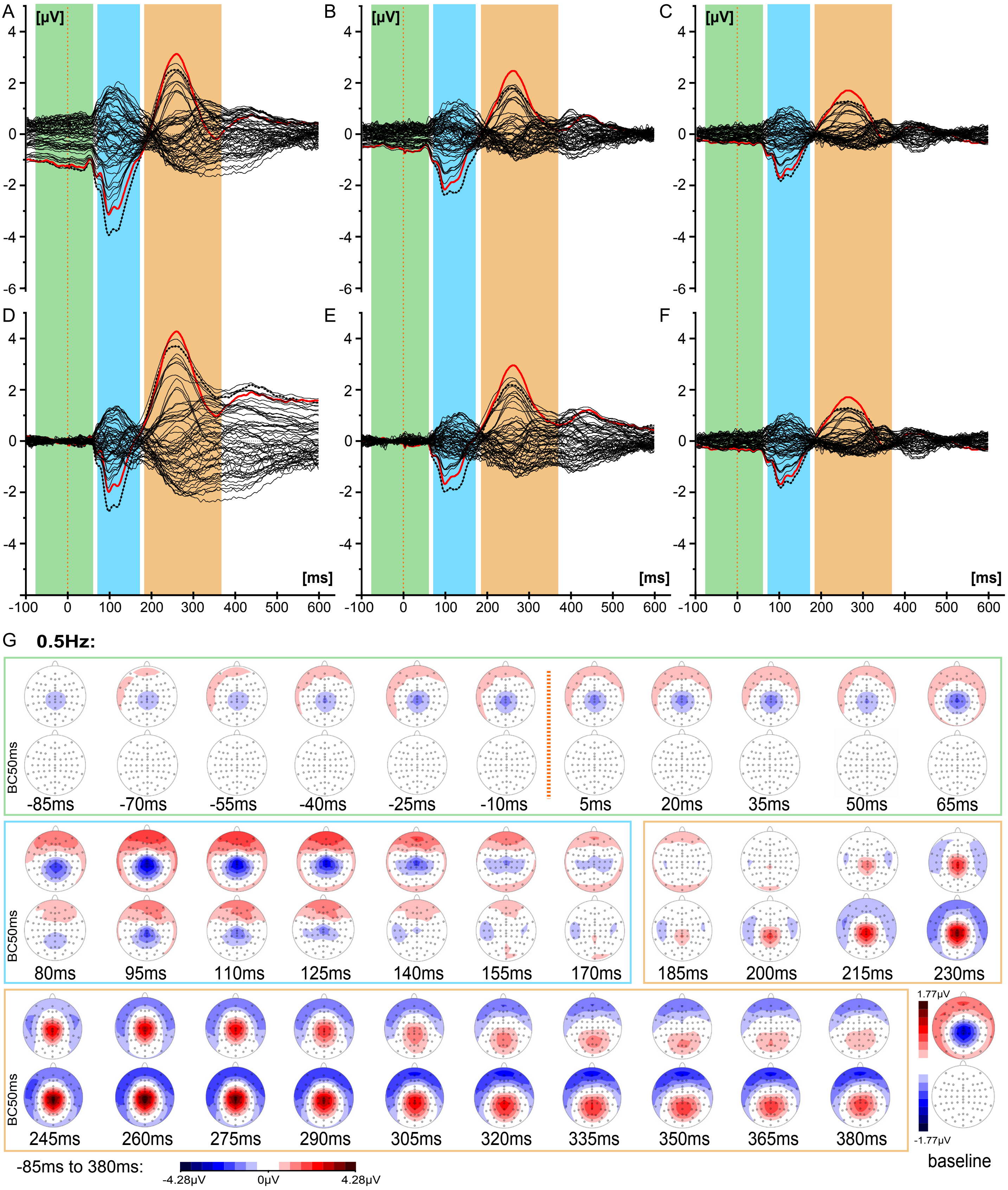

A
B
C
D
E
F
[μV]
[ms]
G
0.5Hz:
BC50ms
-85ms
-70ms
-55ms
-40ms
-25ms
-10ms
5ms
20ms
35ms
50ms
65ms
80ms
95ms
110ms
125ms
140ms
155ms
170ms
185ms
200ms
215ms
230ms
245ms
260ms
275ms
290ms
305ms
320ms
335ms
350ms
365ms
380ms
1.77μV
-1.77μV
baseline
-85ms to 380ms:
-4.28μV
0μV
4.28μV

H **1.1Hz:**

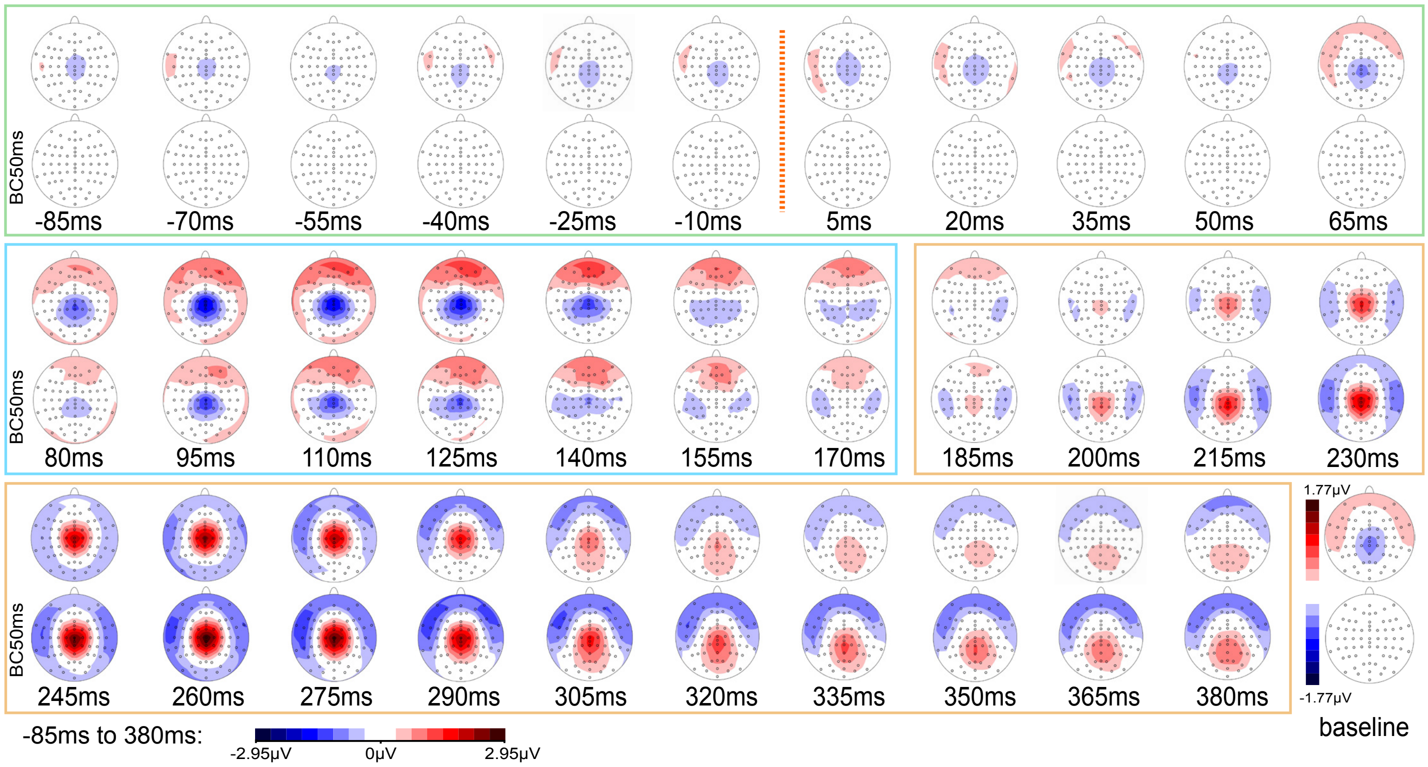

I **1.6Hz:**

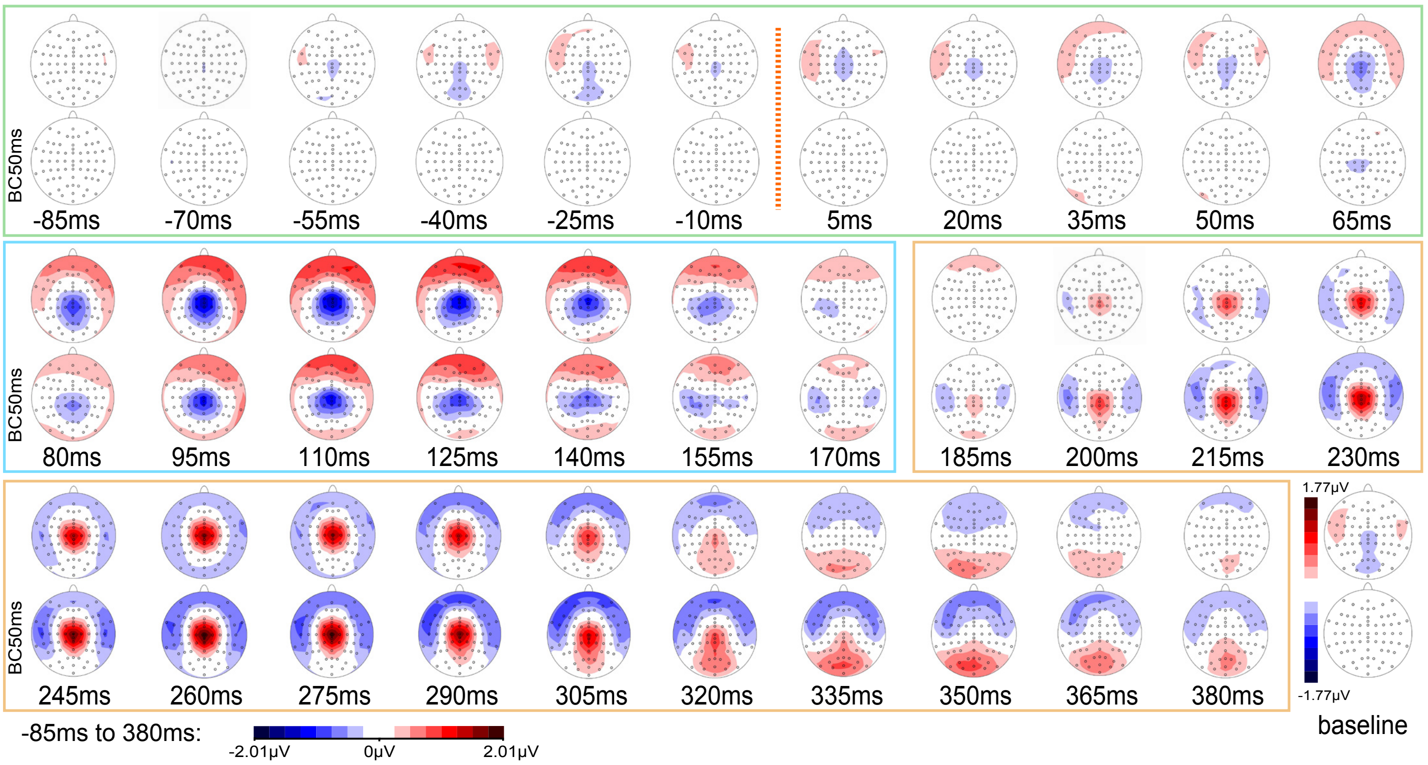

Fig. ESM4 Butterfly plots and time courses of EEG topographies for the location distal urethra. Butterfly plots (-100 to 600ms) are shown for non-baseline corrected (**a-c**) and baseline corrected data (**d-f**) of the three stimulation frequencies (0.5Hz: left, 1.1Hz: middle, 1.6Hz: right, for all: average of 20 subjects). The recordings from Cz-Fz are indicated by the black dashed line and from Cz-AvgRef by the red line. Time courses of topographical maps are shown at 15ms time intervals from -85 to 380ms (average of 20 subjects) for non-baseline corrected data and baseline corrected data of the three stimulation frequencies (**g-i**). The maps are subdivided into three sections: -85 to 65ms (includes pre-stimulus, P1) framed in light green; 80 to 170ms (N1) framed in light blue; 185 to 380ms (P2) framed in orange. The time point of the stimulus is indicated by an orange dotted line. The last map represents the mean topography of the baseline time window (-53 to -3ms) for non-baseline and baseline corrected data

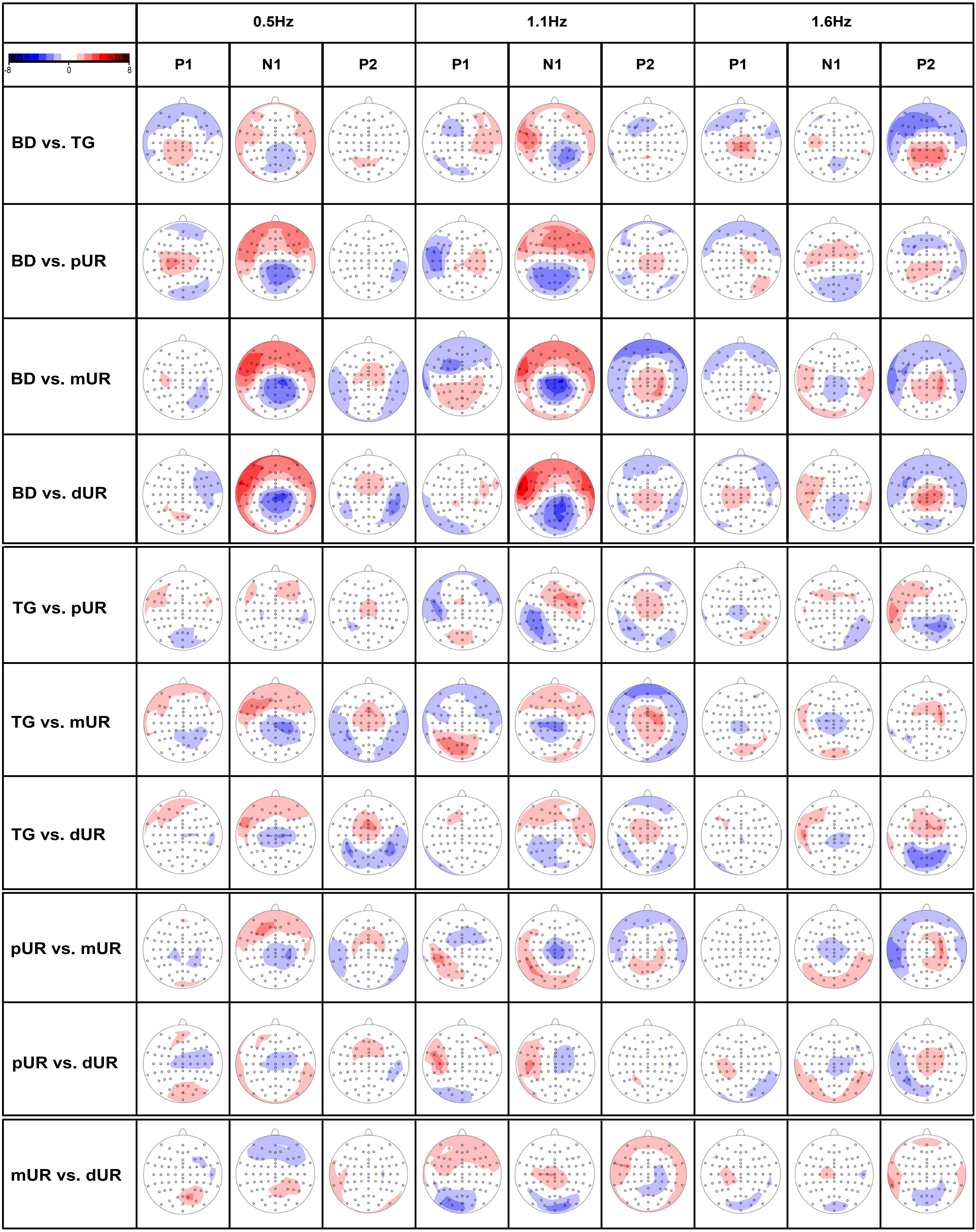
0.5Hz
1.1Hz
1.6Hz
-8
0
8
P1
N1
P2
P1
N1
P2
P1
N1
P2
BD vs. TG
BD vs. pUR
BD vs. mUR
BD vs. dUR
TG vs. pUR
TG vs. mUR
TG vs. dUR
pUR vs. mUR
pUR vs. dUR
mUR vs. dUR

Fig. ESM5 Topographic location comparisons of LUTSEPs. Location effects are shown separately for all stimulation frequencies and the three LUTSEP peaks. Scales of the corresponding topographic maps in t-values (scale from: -8 to +8). Please note that t-values of second color grade represent a statistical trend or significance. BD: bladder dome; dUR: distal urethra; mUR: membranous urethra; pUR: proximal urethra; TG: trigone

Supplementary Results

<u>Pudendal SEP Results:</u>

Single channel analyses revealed for pudendal nerve stimulation significant differences between the two marker sets for the negative component N50 (Table 2 & 3).

LMMs revealed a significant gender effect with men showing longer pudendal SEP latencies compared to women. Hence, we performed a gender-specific analysis of the pudendal SEP data. Body height had no significant effect on latencies, while positive relationship between BMI and P40/N50 latencies were shown.

Butterfly plots and topographical maps of the pudendal nerve stimulation along the entire segment are shown in supplementary Fig. 6.

Statistical analyses showed consistent neural activation along the entire pudendal SEP segment (-100-200ms, except between -50 and -30ms) and for all marker positions (P40, N50, P65, N85) of Cz-Fz and Cz-AvgRef recordings. TANOVA revealed that different sources were active between women and men in the time period of about 45ms to 90ms after stimulus, while no significant differences in map strength were shown along the entire segment (supplementary Fig. 7a).

The topographic pattern analysis on the between-subject factor gender yielded six representative topographic scalp maps (supplementary Fig. 7b). The remaining analyses were consequently based on 6 (1–6) microstate classes, respectively. Their

temporal occurrences are shown in supplementary Fig. 7c accompanied by the corresponding SEP grand averages (supplementary Fig. 7d).

The results of the time-locked components in the entire SEP segment were confirmed by the individually set marker positions, showing different brain function states between women and men for the N50, P65, N85 marker (supplementary Fig. 7e). The topographical maps including the t-maps illustrating these differences between women and men are shown in supplementary Fig. 7f.

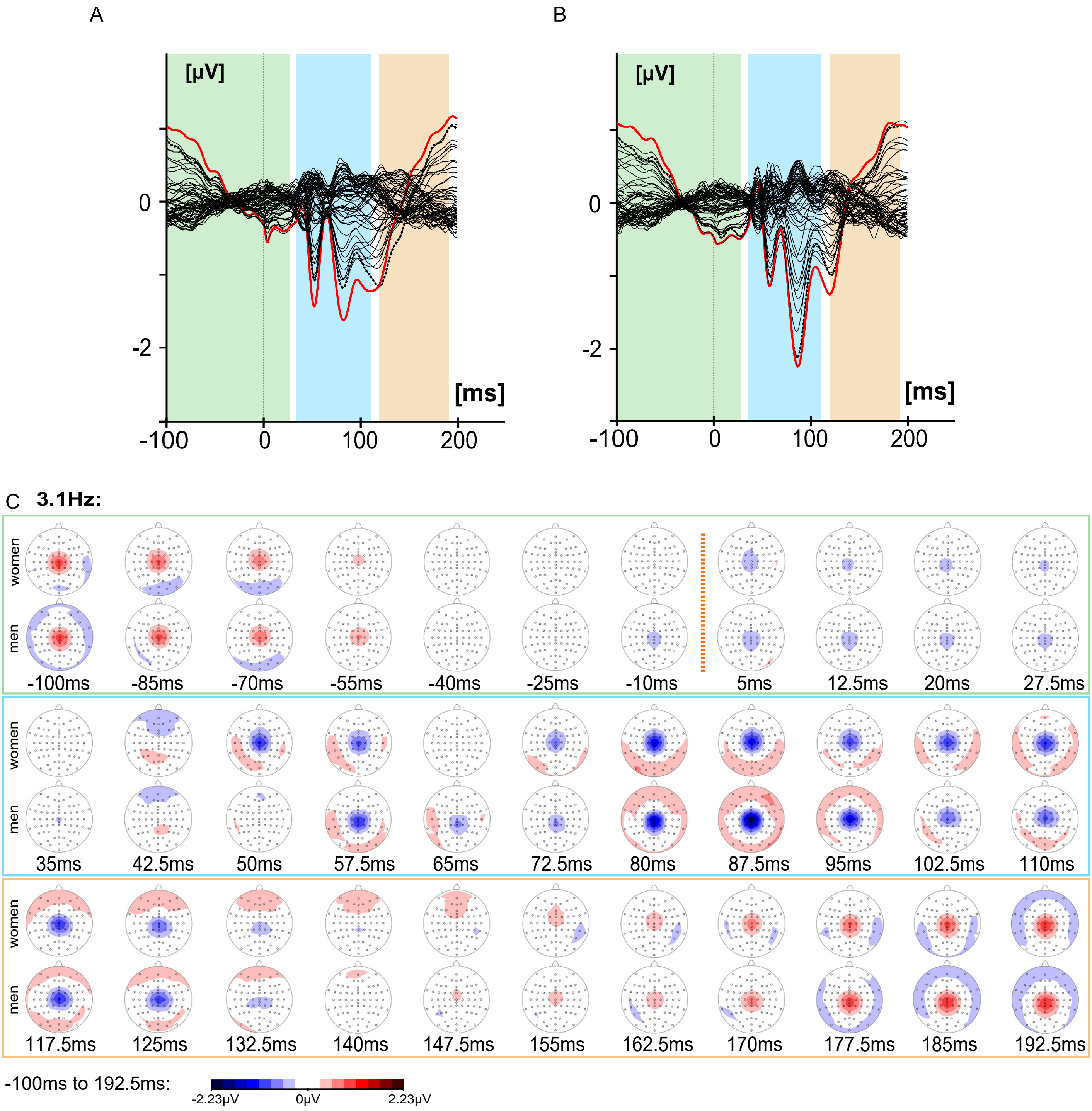

Fig. ESM6 Butterfly plots and time courses of EEG topographies for pudendal nerve stimulation. Butterfly plots (-100 to 200ms) of women (**a**, average of 40 subjects) and men (**b**, average of 50 subjects) are shown for non-baseline corrected data. The recordings from Cz-Fz are indicated by the black dashed line and from Cz-AvgRef by the red line. Time courses of topographical maps are shown at 15ms/7.5ms time intervals from -100 to 192.5ms (average of 90 subjects) for non-baseline corrected data (**c**). The maps are subdivided into three sections: -100 to 27.5ms framed in light green; 35 to 110ms framed in light blue; 117.5 to 192.5ms framed in orange. The time point of the stimulus is indicated by an orange dotted line

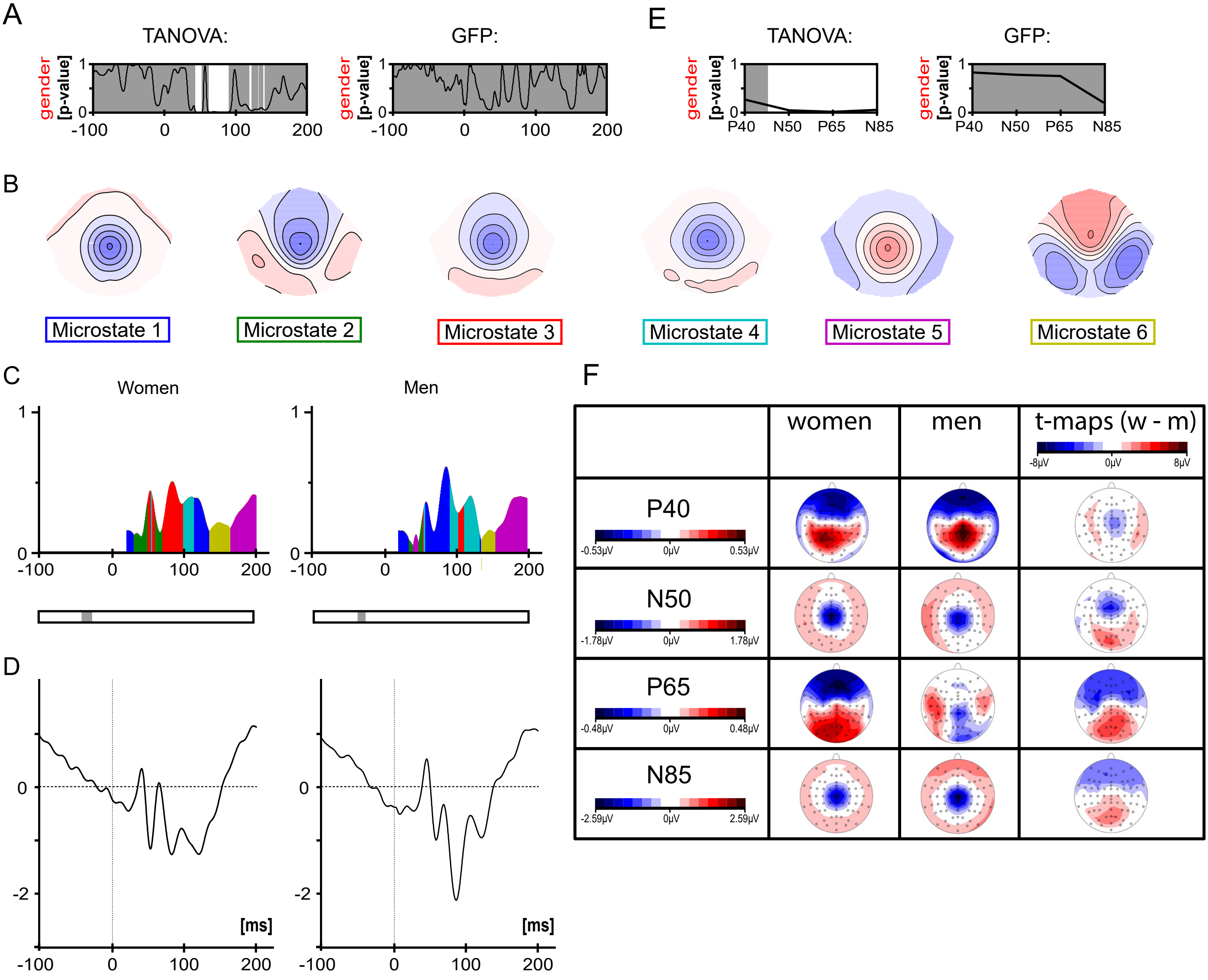

Fig. ESM7 Results of the topographical analysis obtained from non-baseline-corrected pudendal data and the corresponding wave shapes. **a** TANOVA (left) and GFP results (right, n=83; factor: gender) are shown along the entire segment (-100 to 200ms). The black line marks the p-value as a function of time (x-axis). Grey areas indicate non-significant time intervals while the white areas indicate significant periods between topographic maps of the factor gender. **b** The six microstate maps obtained from the cross-validation procedure are labelled from 1 to 6 and displayed in sequence of occurrence from left to right (n=83; orientation: nose up, right is right). Different colors are attributed to different microstate classes. **c** The time course of each microstates map is shown as a function of GFP for the women and men. Below, results of the topographic consistency test are shown (grey: not significant; white: $p<0.05$). **d** Grand average cortical SEPs derived from Cz-Fz in response to pudendal nerve stimulation. **e** TANOVA (left) and GFP (right, n=83; factors: gender) are shown for the four Cz-Fz marker positions. The black line marks the p-value for P40, N50, P65 and N85 peak amplitudes (x-axis). Grey areas indicate non-significant time intervals while the white areas indicate significant periods between topographic maps of the factor gender. **f** Mean topographical maps are displayed for women (n=35) and men (n=48) for P40, N50, P65, N85 based on the individually set Cz-Fz markers. T-maps illustrating gender effects are shown based on the individually set Cz-Fz marker positions. Scales of the corresponding topographic maps in µV/t-values, respectively. Second color grade represents significance. GFP: global-field power; m: men; SEP: sensory evoked potential; w: women
